# Unraveling the genetic evolution of SARS-CoV-2 Recombinants using mutational dynamics across the different lineages

**DOI:** 10.3389/fmed.2023.1294699

**Published:** 2024-01-15

**Authors:** Varsha Ravi, Uzma Shamim, Md Abuzar Khan, Aparna Swaminathan, Pallavi Mishra, Rajender Singh, Pankaj Bharali, Nar Singh Chauhan, Rajesh Pandey

**Affiliations:** ^1^Division of Immunology and Infectious Disease Biology, INtegrative GENomics of HOst-PathogEn (INGEN-HOPE) Laboratory, CSIR-Institute of Genomics and Integrative Biology (CSIR-IGIB), Delhi, India; ^2^CSIR-Central Drug Research Institute, (CSIR-CDRI), Lucknow, Lucknow, India; ^3^CSIR-North East Institute of Science and Technology (CSIR-NEIST), Jorhat, Assam, India; ^4^Department of Biochemistry, Maharshi Dayanand University, Rohtak, India; ^5^Academy of Scientific and Innovative Research (AcSIR), Ghaziabad, India

**Keywords:** SARS-CoV-2, lineages, mutations, recombination, linkage disequilibrium, breakpoints

## Abstract

**Introduction:**

Recombination serves as a common strategy employed by RNA viruses for their genetic evolution. Extensive genomic surveillance during the COVID-19 pandemic has reported SARS-CoV-2 Recombinant strains indicating recombination events during the viral evolution. This study introspects the phenomenon of genome recombination by tracing the footprint of prominent lineages of SARS-CoV-2 at different time points in the context of on-going evolution and emergence of Recombinants.

**Method:**

Whole genome sequencing was carried out for 2,516 SARS-CoV-2 (discovery cohort) and 1,126 (validation cohort) using nasopharyngeal samples collected between the time period of March 2020 to August 2022, as part of the genomic surveillance program. The sequences were classified according to the different lineages of SARS-CoV-2 prevailing in India at respective time points.

**Results:**

Mutational diversity and abundance evaluation across the 12 lineages identified 58 Recombinant sequences as harboring the least number of mutations (*n* = 111), with 14 low-frequency unique mutations with major chunk of mutations coming from the BA.2. The *spontaneously/dynamically increasing* and *decreasing* trends of mutations highlight the loss of mutations in the Recombinants that were associated with the SARS-CoV-2 replication efficiency, infectivity, and disease severity, rendering them functionally with low infectivity and pathogenicity. Linkage disequilibrium (LD) analysis revealed that mutations comprising the LD blocks of BA.1, BA.2, and Recombinants were found as minor alleles or as low-frequency alleles in the LD blocks from the previous SARS-CoV-2 variant samples, especially Pre-VOC. Moreover, a dissipation in the size of LD blocks as well as LD decay along with a high negative regression coefficient (R squared) value was demonstrated in the Omicron and BA.1 and BA.2 lineages, which corroborated with the breakpoint analysis.

**Conclusion:**

Together, the findings help to understand the evolution and emergence of Recombinants after the Omicron lineages, for sustenance and adaptability, to maintain the epidemic spread of SARS-CoV-2 in the host population already high in immunity levels.

## Introduction

SARS-CoV-2, a causative agent of COVID-19 disease, emerged in the late 2019 in Wuhan, China (1, 2) and spread worldwide due to its highly contagious form leading to a range of symptoms from asymptomatic to mild-to-severe respiratory illness in the humans (3). As of 14 September 2023, there have been 770,437,327 confirmed SARS-CoV-2 infection cases and 6,956,900 deaths reported globally (4). Extensive genomic surveillance and research on SARS-CoV-2 has provided valuable genomic data, revealing evolutionary events and the emergence of unique variants (5). The interplay between evolution, ecology, and epidemiology was a defining feature of RNA viruses such as coronaviruses (5). Understanding the virus’s evolution can be crucial in predicting future paths, public health preparedness, and developing prevention/treatment strategies (6). Viral evolution is a complex phenomenon, involving a balance between successful replication and transmission (5). Virus mutation rate and replication errors contribute to genetic diversity, providing the basis for selection (5). Recombination, observed in SARS-CoV-2 and other related viruses, combined mutations from different strains which facilitates adaptability (6, 7). During the first 8 months of the SARS-CoV-2 emergence, visible evolution was limited due to factors such as a small global viral population, non-pharmaceutical interventions, limited dissemination, and under-sampling of the virus (5). Initially, it was expected that SARS-CoV-2 would evolve slowly due to the proofreading ability of its polymerase enzyme (8). Hence, earlier studies relying on linkage disequilibrium identified limited instances of viral recombination and evolution (9–14). This has facilitated the emergence of divergent SARS-CoV-2 lineages worldwide, including Alpha, Beta, and Gamma variants of concern (VOCs). These lineages exhibited rapid evolutionary rates and acquired numerous additional genomic mutations. Speculative evidence suggested recombination between Alpha and Delta variants in a limited number of SARS-CoV-2 infections in Japan (15). Emergence of the Omicron variant, characterized by dozens of spike gene mutations, marked a new phase of the COVID-19 pandemic (16). Initially, Omicron sub-lineages BA.1, BA.2, and BA.3 emerged independently in October 2021 (16), but later, BA.2 diversified into BA.2.12.1, BA.2.75, and BA.5, which were phylogenetically distinct from the BA.2 sub-lineages and gained high global frequency (17). Subsequently, BA.4 and BA.5 were identified between December 2021 and January 2022, respectively (18). BA.1 and BA.5 emerged with common mutations in the spike region (N501Y, E484K, ΔH69/V70) despite their different origins from South Africa and Botswana respectively, indicating convergent evolution (13, 19, 20). Thus, the increased genetic diversity of SARS-CoV-2 allowed multiple lineages to co-circulate and aided in the detection of Recombinants (5, 21). Several Recombinant variants have since been reported (22). The XA lineage, a Recombinant variant, was first identified in the United Kingdom (23). Preliminary genomic characterization of the emergent SARS-CoV-2 lineage in the United Kingdom was defined by a novel set of spike mutations (24). In North America, lineage XB (B.1.631/B.1.634) was widespread and additional Recombinant lineages (XD, XF, and XE) which combined different variants were discovered (25). Recombination events likely contributed to the emergence of BA.3, BA.4, and BA.5 lineages too (26).

The future evolutionary trajectory of SARS-CoV-2 remains uncertain, with questions about whether diverse lineages will continue to emerge or if the virus will undergo the transition to a slower adaptation process? Understanding the evolution of Recombinant lineages through the genetic variability patterns was observed since pre-VOC times is crucial for interpreting the outcome of such transitions to sustenance and adaptability or enhanced transmissibility or virulence for reconstructing the epidemic spread.

In this study, we analyzed 2,516 in-house SARS-CoV-2 sequences as a discovery cohort, since Pre-VOC times, to delineate the mutation profiles associated with different lineages and understand the evolutionary trajectory of SARS-CoV-2 in India, leading to the emergence of the Recombinants. Linkage disequilibrium analysis was performed among all the SARS-CoV-2 lineages to evaluate their impact on the fitness and adaptability associated with the Recombinants. Moreover, a causal relation between LD decay and breakpoint analysis revealed the propensity of Omicron lineages and Recombinants to undergo evolution for enhanced sustenance in the host population. We parallelly validated this framework of analysis in a separate data set of 1,126 SARS-CoV-2 genomes as the validation cohort from the different Indian states to capture the differential traits of the evolution of the SARS-CoV-2 virus.

## Materials and methods

### SARS-CoV-2 whole genome sequencing

The SARS CoV-2 whole genome sequencing was performed using Oxford Nanopore and Illumina Sequencing platforms. Library preparations have been carried out using standard protocols and guidelines of the Illumina COVIDSeq (Cat. No.20043675 and reference guide: 1000000126053 v04) and Oxford Nanopore Rapid barcoding kit (SQK-RBK110.96). The sequencing methodology and analysis pipeline of both platforms have been previously published (27).

### Sample segregation

Sequenced data for COVID-19-positive patients were taken from the SARS-CoV-2 genomic surveillance program between the time period of March 2020 and August 2022. A total of 3,642 genome sequences were considered for analysis which had at least 90% SARS-CoV-2 genome coverage and 100X sequencing depth. The FASTA sequences obtained from the genomic analysis were uploaded in Nextclade to obtain lineage-wise classification of the SARS-CoV-2 genome sequences (28).

### Mutational analysis and categorization

VCFs obtained from genomic analysis of lineages were merged using bcftools (29). In order to capture low-frequency mutations, all mutations present in more than one sample in a lineage were considered for mutational analysis. Furthermore, the relative frequencies of mutations in different lineages were estimated by taking into account the sample numbers and presented as a percentage. Seven groups were categorized: Pre-VOC (B.1); Alpha (B1.1.7); Beta (B.1.351); Delta and Delta plus as Delta (B.1.617.2); BA.1, BA.2, and Recombinant. For each lineage, from pre-VOC to Recombinant, low, medium, and high-frequency mutations were distributed as low (10% frequency and below), medium (11–50% frequency), and high (above 50%).

### Linkage disequilibrium (LD) analysis

Variants identified in seven different lineages with minor allele frequencies (MAF) ≥0.05% were used to estimate the LD. To perform haplotype block analysis and detect the recombination event, we used the solid spine of LD option from Haploview (30), which selects the criteria of the first and last mutation to be in strong LD and allows intermediate mutations within to have optimal r^2^ values (31). The selected blocks were intact, where the average r^2^ of all mutations in the block was ≥0.5. We found exceptions in block 1 of pre-VOC, Delta and blocks 1 and 2 in Beta. The plink files for haploview were created using plink commands (32) “plink–out” for changing VCF to the .ped format, and “plink–recode” command was used to get .info and .ped files.

### Detection of LD decay

**Figure 1:**
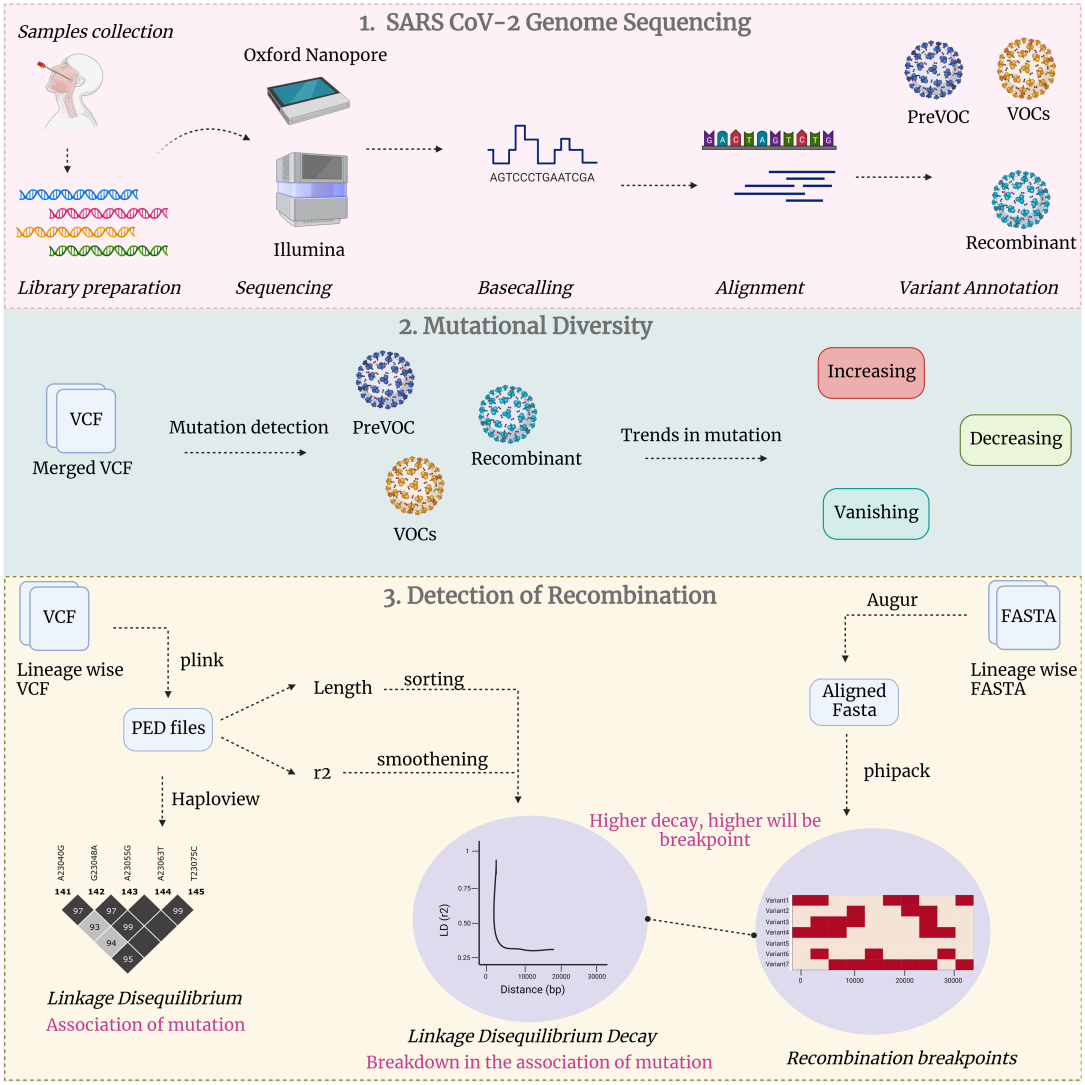
Comprehensive study methodology in three broad categories: Unveiling SARS CoV-2 genome sequencing, exploration of mutational diversity and detecting recombination events.

To estimate the potential gradual breakdown of non-random association of the mutations, LD decay within each lineage from pre-VOC to Recombinant was analyzed. LD decay curves are graphical representations of LD, illustrating how it changes as a function of the physical distance between each pair of SNPs (12). LD decay was accomplished by computing the difference between genetic distances as LD2 (Position) minus LD1 (Position) and its corresponding LD (*r*^2^). Preceding decay, the data points were smoothened by a locally estimated scatterplot smoothing (LOESS) algorithm. This non-parametric regression technique fitted a smooth curve to the sorted distances and their corresponding LD values, effectively capturing underlying patterns in the data. Furthermore, a horizontal threshold line was introduced to the plot at an LD value of 0.1. This threshold served as a visual marker, aiding in the identification of the point at which LD is deemed to have significantly decayed. The data points displayed in the graph offered a tangible representation of LD values at varying genetic distances. To quantify the connection between genetic distance and LD decay, the coefficient and R-squared values were calculated using the statsmodel (33) library in Python version 3.11.4. The coefficient states whether the distance and LD (r2) were positively or negatively correlated and R-square determined the coefficient regarding how much the independent variable (distance) could be explained by the dependent variable (LD), with a larger R-squared value denoting better regression Figure 1 illustrates the comprehensive methodology utilized for carrying out this study.

### Identification of recombination breakpoints using PhiPack

Potential recombination breakpoints (regions where recombination events might have occurred) were detected using the Pairwise Homoplasy Index (PHI). FASTA files were generated for the variant data of all the lineages from the Pre-VOC to Recombinants, and multiple sequence alignment was performed separately for all the lineages. Furthermore, we employed the “Profile” feature of the PhiPack tool to detect the recombination breakpoints and the refined incompatibility matrix (34, 35). We configured the profile analysis with default parameters for robust statistical outcomes. The window size was ascertained based on the fraction of the total samples and implemented with 1,000 permutation tests. Within the framework of PhiPack, the null hypothesis centered on the absence of recombination events, whereas the alternate hypothesis acknowledged the plausible occurrence of recombination events (36). The resultant *p-values* below the established threshold of 0.05 were considered as recombination incidences (37).

## Results

### Mutational landscape across different lineages of SARS-COV-2 from pre-VOC to Recombinants

**Figure 2:**
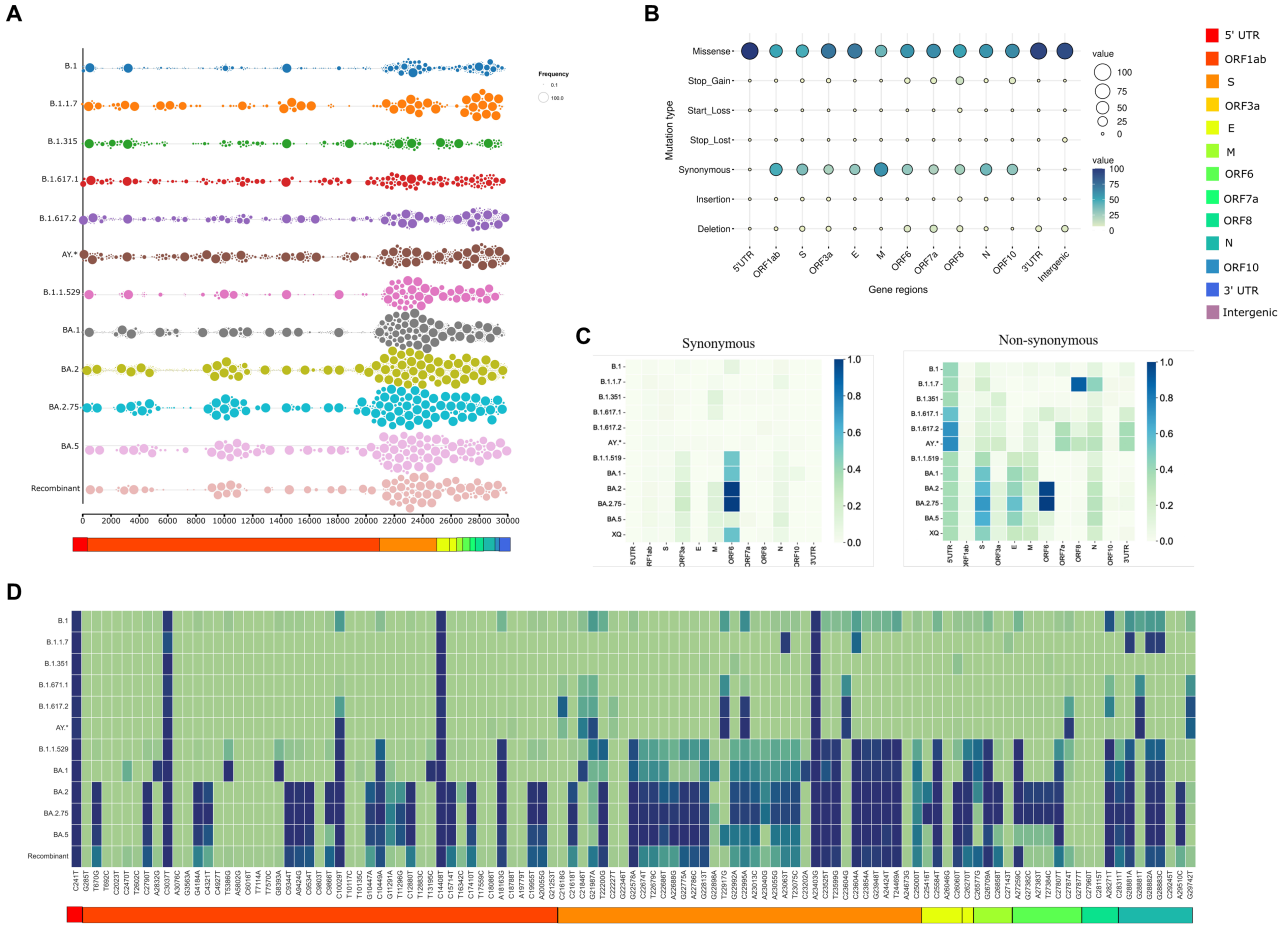
Trends of SARS CoV2 mutational dynamics with respect to the Recombinant variants. **(A)** The plot represents the mutational landscape of the 12 lineages from the Pre-VOC (B.1) to the Recombinant across the SARS-CoV-2 genomes. **(B)** Types of mutations harboring different SARS-CoV-2 gene regions. **(C)** Relative abundance of the synonymous and non-synonymous mutations across different SARS-CoV-2 gene regions. **(D)** Heatmap shows the tendency of mutational evolution from the Pre-VOC to the Recombinant.

The inhouse SARS-CoV-2 genome surveillance program enabled us to gather 2,516 sequences from the time period of March 2020 to August 2022. These sequences were obtained using a coverage exceeding 90% of SARS-CoV-2 genome with a sequencing depth of minimum 100X. They were further categorized according to the lineage, leading to the identification of 12 lineages. B.1 was termed pre-VOC and had *n* = 273 samples, B.1.1.7 (Alpha; *n* = 30), B.1.351 (Beta; *n* = 49), B.1.617.1 (Kappa; *n* = 29), B.1.617.2 (Delta; *n* = 236), and AY* (Delta-plus; n = 65). Omicron was segregated into five sub-lineages: B.1.1.529 (*n* = 268), BA.1 (*n* = 610), BA.2 (*n* = 819), BA.2.75 (*n* = 64), and BA.5 (*n* = 15). We identified 58 sequences as Recombinants wherein XAP lineage had the largest number of genomes (*n* = 44), followed by XT (*n* = 6). Two sequences each belonged to the Recombinants—XM, XAE, XQ, and XU, respectively. Sequencing metadata and sample information are tabulated in Supplementary Table S1. Collating the mutation data, we identified 3,056 unique mutations. Figure 2A represents the mutational landscape of all the 12 lineages across the SARS CoV-2 genomes from the pre-VOC to Recombinants. The mutational region in the SARS-CoV-2 genome has been observed to be in the 3’end region, downstream of ORF1ab (essentially involved in virus replication and reproduction) across all the lineages since the Pre-VOC times, demonstrating the importance of the ORF1ab region toward providing stability and functional edge to the virus toward its sustenance in the human host population. Moreover, an overall mutational surge in the 3′ region was observed from the Delta-plus (AY*) lineage onwards although a burst in mutations occurred in the spike region of B.1.1.529 (Omicron), following a similar trend for all the other lineages of Omicron.

Indeed, this mutation crowd started decreasing in the Recombinants. Furthermore, mutation-type analysis across the SARS-CoV-2 genome/gene regions deliberated a heightened presence of missense and synonymous mutations, followed by deletions. The remaining part consisted of start/stop gain and loss (leading to truncated ORFs), insertions as well as mutations in the untranslated regions. Missense dominates the different SARS-CoV-2 gene regions except for the M region, which is taken over by synonymous mutations. The ORF8 gene region had a good proportion of all the types of mutations, which coincides with its proposed functionality toward within-individual fitness only [e.g., viral replication and immune evasion), but neutral or even disadvantageous for transmission in the population (38)]. Deletions were higher in the ORF7a, wherein these mutations could alter viral-host interactions and immunomodulatory features of the SARS-CoV-2 virus. Segregation of mutation types across the lineages identified the highest percentage of missense mutations in B.1.1.529, whereas synonymous mutations were more in BA.2 and deletions were higher in B.1.617.1 (Kappa) (Supplementary Figure S1). Furthermore, we looked into gene-wise mutation abundance (overall prevalence of mutations in specific gene regions which is “mutation per sequence”) across the different lineages as demonstrated in Figure 2C. For synonymous mutations, relative abundance was only observed for the ORF6 region and that too in the Omicron lineages, whereas the non-synonymous gene regions differentially occurred among the lineages. Mutational abundance dominated ORF8 and nucleocapsid in Alpha, 5’ UTR in Delta. Omicron lineages highlighted the Spike, Envelope, and ORF6 gene regions. Interestingly, the Recombinants showed relative mutational abundance of the synonymous mutations similar to BA.1 and B.1.1.529, whereas the non-synonymous mutations were diluted across the genome with moderate abundance in the Spike. This led us to delve into mutations captured across the Recombinants. Interestingly, across all the 12 lineages, Recombinants harbored the least number of mutations (*n* = 111) across the 58 Recombinant sequences. Looking at the frequency of these mutations across all the 12 lineages, Figure 2D highlighted four SNVs, C241T (5’UTR), C3037T (ORF1ab: F924F), C14408T (ORF1ab: P4715L), and A23403G (S: D614G), with strong allelic associations. This was indispensably associated with the SARS-CoV-2 genomes since the Pre-VOC times. Fourteen unique low-frequency mutations were identified with equal distribution of the synonymous and non-synonymous variants. Notably, out of the 47 mutations of ORF1ab, 24 mutations were from BA.2, 9 from B.1.1.529, 4 from BA.1 and 10 were unique. Ten high-frequency mutations of the Delta (B.1.617.2) variant with reported functional relevance [ORF1ab: T3255I, S: T19R, S: T95I, S: G142D, S: L452R, S: T478K, S: P681R, C27874T (Intergenic), N: R203M and G29742T (3’UTR)] were also found in the Recombinants (Supplementary Table S2). Surprisingly, these mutations from the Delta (B.1.617.2) were present in low frequency in the Recombinants.

### SARS-CoV-2 Recombinant emergence ascertained through mutational dynamics across the distinct lineages during the course of evolution

**Figure 3:**
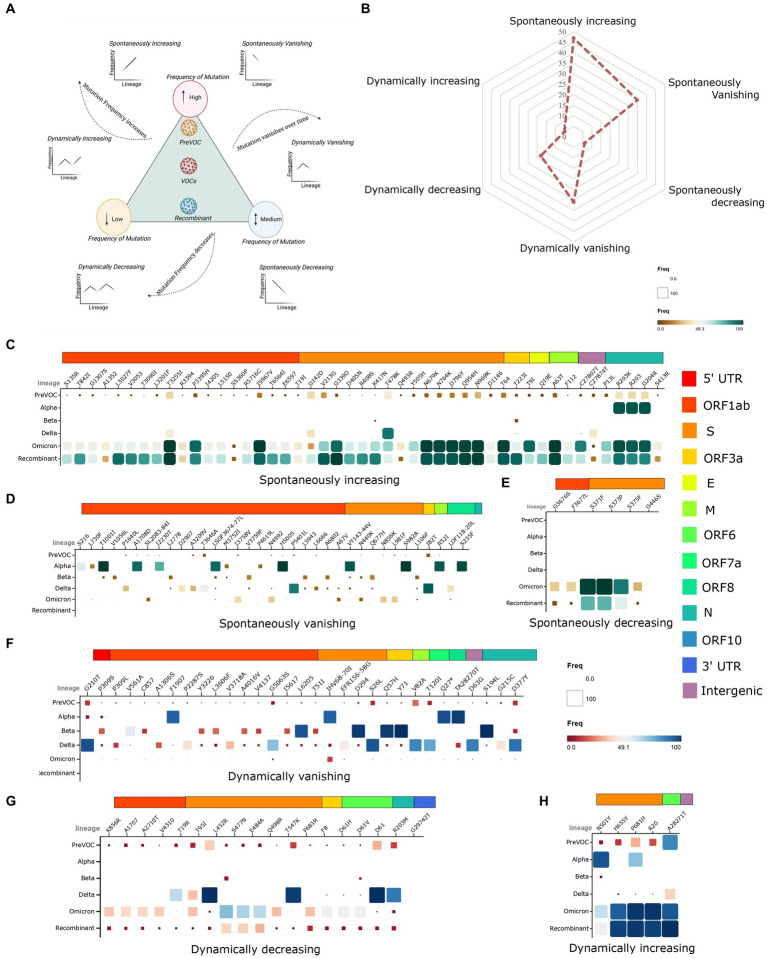
Evolutionary trends of mutations from the Pre-VOC to the Recombinant. **(A)** Graphical representation of the low, medium, and high-frequency mutations, depicting increasing, decreasing, and vanishing trends in the two categories of spontaneous and dynamic occurrence. **(B)** Spider plot illustrating the overall presentation of mutations falling in each of the six study category types. **(C–E)** Matrix plot of spontaneously increasing, vanishing, and decreasing mutations from the Pre-VOC to the Recombinant. **(F–H)** Matrix plot of dynamically vanishing, decreasing, and increasing mutations.

As the mutational landscape underlying the viral genome architecture played a significant role in viral evolution, we looked into the mutational pattern across the lineages from the Pre-VOC to the Recombinants. Primarily, all the lineages were grouped according to their emergent/dominant time points that were classified into six study groups: Pre-VOC (B.1), Alpha (B.1.1.7), Beta (B.1.351), Delta [Kappa (B.1.617.1), Delta (B.1.617.2) and Delta-plus (AY.*)], Omicron (B.1.1.529, BA.1, BA.2, BA.2.75, and BA.5), and Recombinant. Subsequently, individual mutations were divided into three categories based on their frequencies present in each study group—low (10% and below), medium (11–50%), and high (50% and above). Furthermore, the pattern of mutational frequencies (low, medium, and high) throughout the time points (lineages) was analyzed to understand the viruses’ selection during their evolution. Resultantly, it demonstrated six mutational trends: a) dynamically increasing, b) dynamically decreasing, c) dynamically vanishing, d) spontaneously increasing, e) spontaneously decreasing, and f) spontaneously vanishing (Figure 3A). The increasing vs. decreasing trend was identified by taking their frequencies at the Recombinant time points into account when compared to the rest of the lineages. The mutational abundance in each of the study groups was illustrated through a spider plot (Figure 3B). We observed the highest number of mutations (*n* = 47) in the *spontaneously increasing* category, wherein frequencies were seen to be high in the Recombinant but low in the Pre-VOC as well as other variants (Figure 3C). These mutations acquired during the Pre-VOC time became majorly dominant in the Omicron and were further passed on to the Recombinants. Few mutations of this category, such as S: N679K (T23599G), S: N764K (C23854A), S: Q954H (A24424T), and S: N969K (T24469A), reportedly led to the loss of protein function, thereby decreasing SARS-CoV-2 infectivity, replication, and disease severity (39–41). In the *spontaneously vanishing* (*n* = 36), we observed high-frequency mutations in the Alpha, which were found to vanish or decrease afterward, resulting in complete de-selection by the virus during the Recombinant emergence (Figure 3D). Notably, the mutations S: N440K (T22882G) and S: S982A (T24506G) in the Alpha variant were reported to be significantly associated with enhanced viral entry and reduced antibody neutralizations, which were absent in the Recombinants (42). Strikingly, we observed only six mutations as *spontaneously decreasing* (Figure 3E), which majorly arose in the Omicron and were retained in the Recombinants. Looking at the dynamic mutations, the vanishing category harbored the highest mutations (*n* = 31), which showed complete absence in the Recombinants with low/medium frequency presence in the Pre-VOC and varying frequency trends in the other lineages (Figure 3F). Similarly, *dynamically decreasing* mutations demonstrated low frequency in the Recombinants with high/medium/low presence in the previously arisen variants (Figure 3G). Notably, dynamically vanishing and decreasing mutations were principally present in the Beta and Delta and were eliminated by the virus till it evolved into Omicron with a similar trend in the Recombinant. Interestingly, such high-frequency mutations of Delta such as S: L452R (T22917G), S: P681R (C23604G), and N: R230M (G28881T) are reported to functionally elevate the SARS-CoV-2 infection and replication (43–46), which were absent in the Omicron and the Recombinant. Other mutations belonging to this category were mutations of the N protein, N: D63G (A28461G) and N: D377Y (G29402T), which were also reported to be significantly associated with ICU admissions (47). Contrarily, the dynamically increasing (Figure 3H) category captured mutations that were essentially present in high frequency in the Alpha/Pre-VOC and subsequently retained in the Omicron/Recombinant with negligible presence in the Delta and Beta. Overall, it is important to note that the mutations associated with the Delta severity demonstrated a reduction in frequency in the Recombinant as well as an overlapping trend with the Omicron highlighting the emergence of the Recombinants through the Omicron lineages.

### Omicron lineages paved the way for the evolution of Recombinants by dismantling the LD blocks

**Figure 4:**
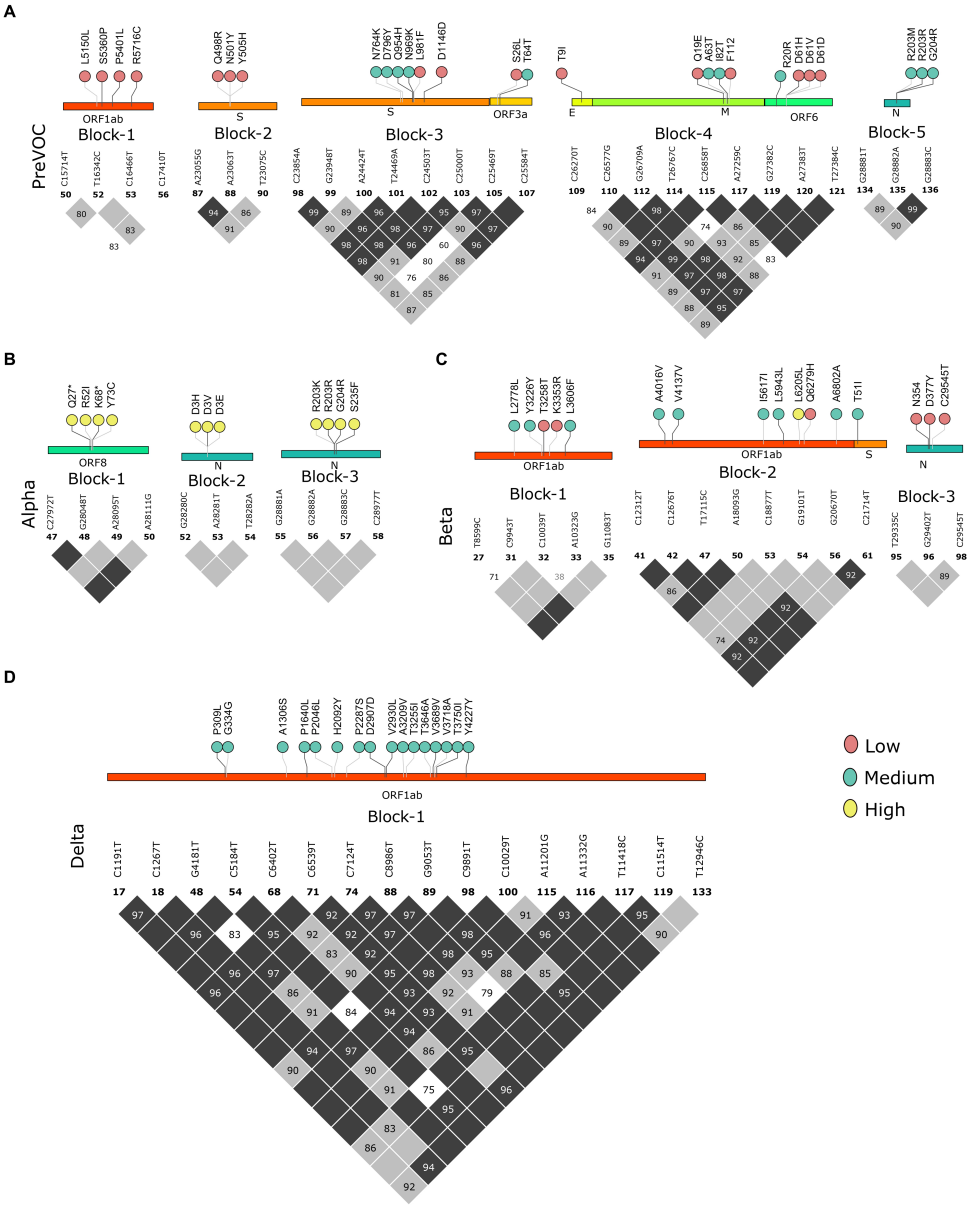
Block-wise information for the Pre-Omicron lineages analogous with the amino acid mutations in the lollipop. LD blocks of **(A)** Pre-VOC, **(B)** Alpha, **(C)** Beta, and **(D)** Delta. Color of the lollipop determines frequency of the mutations in their respective lineages.

Multiple mutational acquisition/depletion trends observed among the different lineages of SARS-CoV-2 furthered our quest to understand the role of linkage disequilibrium (LD). LD analysis has been used to infer evolutionary features and reveal the trends of existing LD blocks in the SARS-CoV-2 genomes along with their dominant mutations since the Pre-VOC times. We analyzed LD blocks for all the lineages stated in the previous result, plus two lineages of Omicron (BA.1 and BA.2), since most of the Recombinant lineages arose from these two lineages of Omicron. Pre-VOC carried five LD blocks, wherein blocks 1, 3, and 4 spanned >1 kb region. Block 1 was constituted by the ORF1ab region, whereas block 3 spanned across the Spike-ORF3a and block 4 across the E-M-ORF6 gene region. The rest of the blocks (blocks 2 and 5) were smaller and spanned the Spike and N gene regions (Figure 4A). Similarly, Alpha demonstrated three small LD blocks in the ORF8 and N (Figure 4B) regions. Interestingly, the Beta variant demonstrated three blocks in LD, wherein block 2 with 9.4 kb length spanned the ORF1ab-S region (Figure 4C), and Delta demonstrated one single LD block, which spanned a larger region of approximately 11.7 kb in the ORF1ab region (Figure 4D).

**Figure 5:**
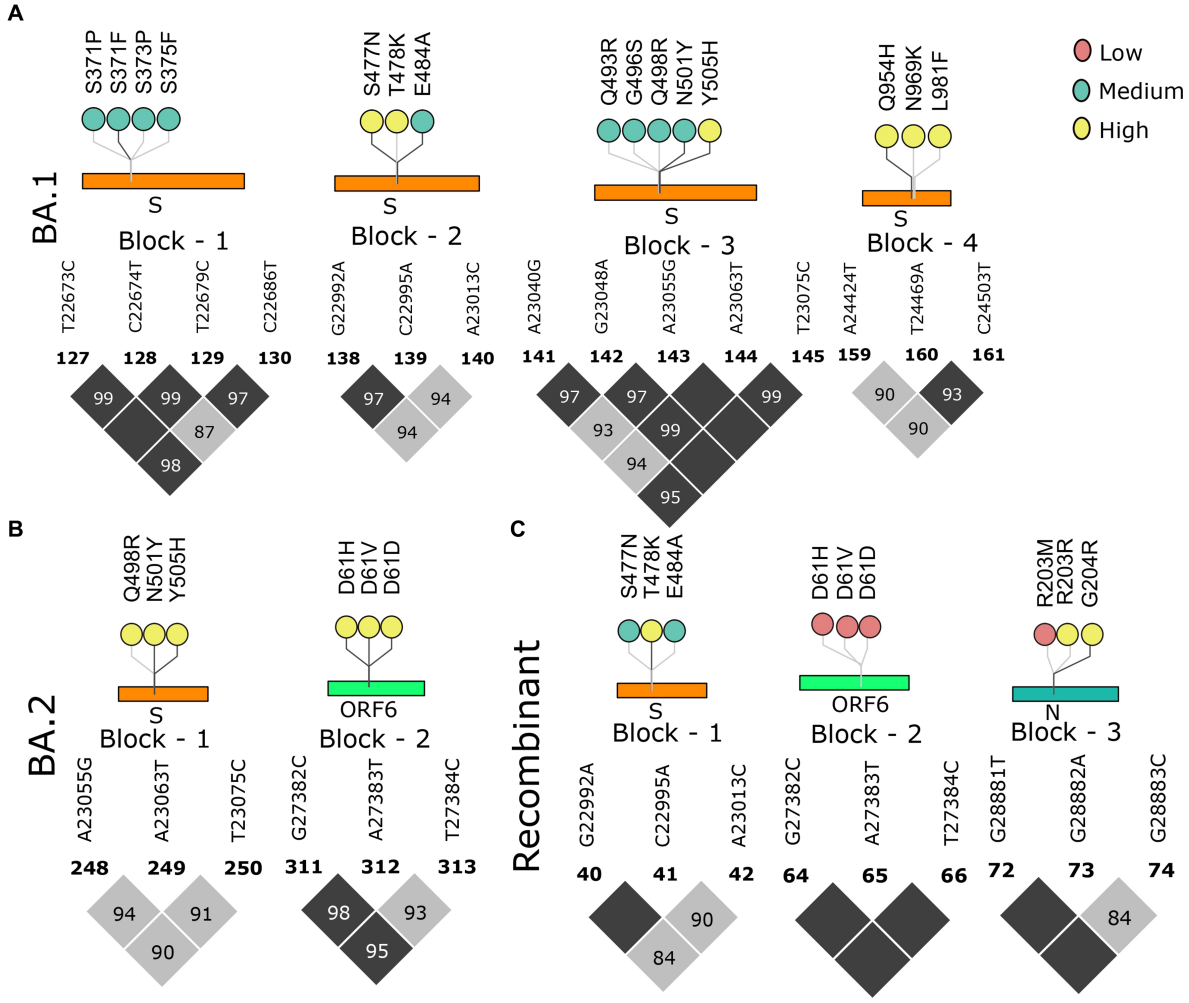
Block-wise information for the Omicron lineage analogous with the amino acid mutations in the lollipop plot. LD block of **(A)** BA.1, **(B)** BA.2, and **(C)** Recombinant. Color of the lollipop determines the frequency of the mutations in their respective lineages.

As we moved toward the Omicron lineages, blocks of strong LD were observed to dissipate leading to the occurrence of smaller blocks, similar to the Pre-VOC in the S, ORF6, and N gene regions. Reducing patterns of LD blocks in the BA.1 and BA.2, leading to the generation of multiple lineages within Omicron suggest a higher propensity for evolution by recombination (Figures 5A,B). Henceforth, the occurrence of Recombinants after the Omicron lineages could be professed/perceived. It is evident that after Omicron lineages, the average r^2^ across the mutations in the block was >0.5 (Supplementary Table S3), indicative of smaller blocks of strong LD being retained. Examining the variants in the LD blocks suggests a clear variation in its frequency between the lineages. Notably, prominent haplotype blocks in the Spike were seen only in the Pre-VOC, BA.1, and BA.2 and were found to be retained in the Recombinant. The mutations were present in low and medium frequency in the Pre-VOC (Figure 4A) which subsequently became high-frequency mutations in the BA.1 and BA.2 (Figure 5B) followed by Recombinants with medium and high frequency (Figure 5C), respectively. Importantly, mutations of the SpikeQ498R/N501Y/Y505H presenting strong LD block in the Pre-VOC (low frequency) were selected in BA.1 (medium and high frequency), along with two other mutations in the Spike region (Q493R/G496S) that reportedly enhanced the immune evasion of BA.1. Subsequently, the same block of Pre-VOC was again selected in the BA.2 (high frequency) but was not retained as the LD block in the Recombinant. Notably, these three reported mutations were associated with increasing viral replication and fitness. However, in the Recombinant, LD block upstream to the positions mentioned above were observed with the mutations, Spike:S477N/T478K/E484A retained from BA.1. Interestingly, S: T478K mutation originated from the Delta (high frequency), whereas S: S477N/E484A was subsequently selected during BA.1 and BA.2 propagations. These mutations were also functionally reported to increase the viral binding with ACE2, increased infectivity, and transmission. It is significant to note that though there was a shift in the LD block to an upstream position, notably all these mutations had a medium and high-frequency representation in the Recombinant. This implies the viral selection toward these positions by either recombination or LD. Another LD block in the ORF6 region, in the Pre-VOC carried D61 low-frequency mutation, which re-emerged in BA.2 with high frequency. D61 protein is instrumental for enhanced viral replication, whereas ORF6: D61V mutation is functionally deleterious with the potential to compromise protein function, viral replication, and indirectly influence the viral immune escape mechanism (48). Subsequently, this LD block was retained in Recombinants but at a very low frequency due to its disruptive function. Thus, we see that many mutations of BA.1, BA.2, and Recombinants were found as minor alleles or as low-frequency alleles in the LD blocks from the previous variant samples, especially the Pre-VOC.

We further looked into the trends of the mutations in the LD blocks (Supplementary Figure S2). Pre-VOC mutations majorly showed an increasing trend as these mutations became a part of the LD blocks retained in the Recombinants. Mutations such as ORF6: D61 in the LD block showed a decreasing trend as they became low-frequency mutations in the Recombinants. Beta and Delta LD blocks consisted of vanishing mutations since none of the LD blocks of the Recombinant coincided with the Delta or Beta. With BA.1 and BA.2, all categories of mutations, increasing, decreasing, and vanishing trends, were noted. The decreasing trend was associated with those LD blocks that are deselected in the Recombinants, such as S: S371/373/375 and S: Q964/N969/L981, and were functionally found to impair or reduce viral infectivity (49). However, another LD block N: R203M/G204R within the Pre-VOC showed opposite trends of N: R203M decreasing while N: G204R increasing in the Recombinants although both mutations were known to increase viral replication (50, 51).

### LD decay and recombination breakpoints align the course of genetic shuffling from the Omicron sub-lineages

**Figure 6:**
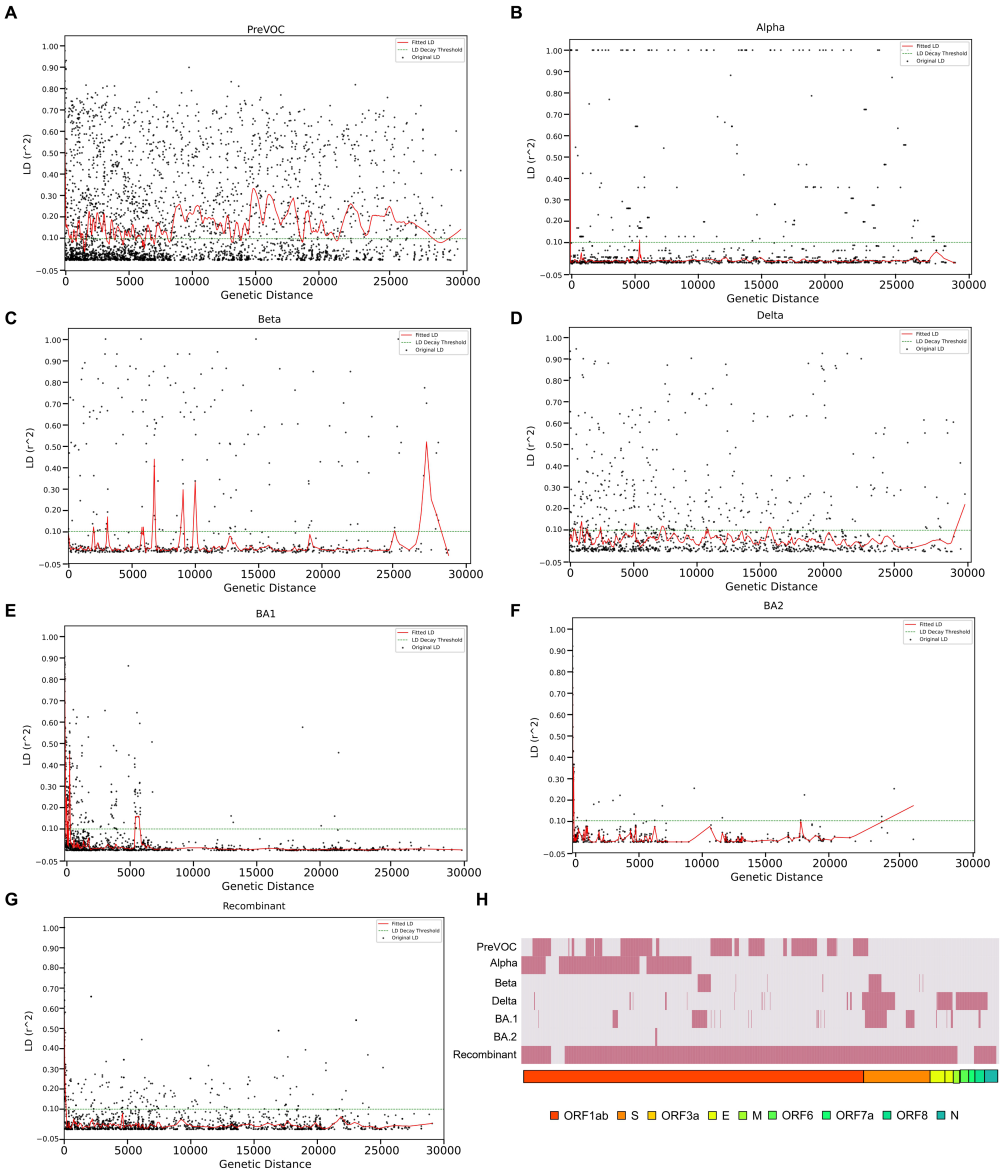
LD decay patterns from the Pre-VOC to the Recombinant. LD decay plot with the fitted original LD. **(A)** Pre-VOC, **(B)** Alpha **(C)** Beta, **(D)** Delta, **(E)** BA.1, **(F)** BA.2, and **(G)** Recombinant. The y-axis denotes LD (*r*^2^) between pairwise combinations of the variant sites, with x-axis denoting distance between these sites. **(H)** Heatmap showing recombination breakpoints from the Pre-VOC to the Recombinant, red signifies breakpoint position.

Linkage disequilibrium decay described how certain mutations that were initially found together in a viral genome can become separated in the course of evolution. This separation is influenced by multiple factors such as the frequency of recombination events during the viral replication, mutation, migration, or admixture, genetic drift as well as the selection pressures acting on the virus. The decline of LD pattern is an indirect method for determining the recombination events wherein higher recombination rate leads to the decay in LD and vice versa (52). After analyzing the LD blocks across the lineages, where majorly, lineages harbored small LD blocks except Delta and Beta, we investigated the decay signal of LD. We plotted LD changes as a function of genetic distance and observed that Alpha, BA.1, BA.2, and Recombinant showed a clear pattern of LD Decay as depicted in Figures 6B,E–G. To delve deeper, we conducted a detailed linear regression analysis between LD (*r*^2^) and distance (between genetic markers). R-square gave a numerical measure of how well the linear regression model captured the change in the dependent variable (LD values) based on the predictor variable (genetic distance). The 0.001 to 0.096 range demonstrated the model’s ability to accurately capture the interplay between the genetic distance and LD values across the different variants. It is interesting to note that the Omicron lineages of BA.1 and BA.2 along with Recombinants stood out with notable negative regression coefficients of −5.834000e-06, −4.410000e-06, −2.174000e-06, respectively, with R-squared value between 0.024 to 0.096 (Table 1), indicative of the factor that increased genetic distance between the mutations which led to decreased LD values (*r*^2^). Moreover, except for Pre-VOC, other lineages including Alpha, Beta, and Delta also showed negative regression coefficients with comparatively lower R-squared value falling between 0.001 to 0.004 (Table 1). Pre-VOC demonstrated a positive correlation coefficient between distance and the LD values along with no evidence of decay after 7,500 genetic distance (Figure 6A).

**Table 1:** Linear regression for distance (L1 & L2) and LD (*r*^2^).

SARS-CoV-2 variants	Co-efficient	R-Square
PreVOC	9.67E-07	0.001
Alpha	−8.60E-07	0.001
Beta	−2.02E-06	0.003
Delta	−1.68E-06	0.004
BA.1	−5.83E-06	**0.096**
BA.2	−4.41E-06	**0.052**
Recombinant	−2.17E-06	**0.024**

Bold values signify substantially higher R^2^ values than the rest of the values.

Decay in BA.1, BA.2, and Recombinants with low regression coefficient directly correlated with the reduced LD block patterns (Figures 5A,B) which suggested its propensity to undergo recombination during evolution. To further explore the recombination events in our data, recombination breakpoints were detected using the PhiPack software. The longest breakpoint position occurred in the Recombinant lineage, spanning nsp3 of ORF1ab to M Gene between the SARS-CoV-2 genomic positions of 3,125–26,875 base pairs (Table 2). Interestingly, ORF1ab regions of Recombinants are expected to be from the BA.2 lineage and S regions from BA.1, confirmed/supported by the clade-defining mutations of BA.1 and BA.2 (53). Furthermore, BA.1 and BA.2 shared few spike region breakpoints with Beta and Delta and ORF1ab regions with Pre-VOC and Alpha. BA.1 breakpoints spanned across almost entire SARS-CoV-2 genome till the M gene region (genomic position 26,500–26,600), whereas BA.2 breakpoints were captured only for the nsp4 region (genomic position 8,600–8,700) of ORF1ab (Table 2). For Pre-VOC and Alpha, breakpoints were identified until ORF1ab and the beginning of the Spike regions, whereas for Beta and Delta, breakpoints were toward the 3’UTR region. This suggests that Alpha and BA.2 preserves 3’UTR and Delta retains 5’UTR during the recombination events (54). Additionally, the variants present in the breakpoint regions were invariably in weak LD with the mutations present in the haploblocks surrounding the breakpoint areas. It was also noted that high-frequency mutations in the breakpoint regions increased by 38% from BA.1.

**Table 2:** Breakpoint information from the SARS-CoV-2 Pre-VOC to Recombinant.

Lineage SARS-CoV-2 variant	Breakpoint position	Total length	No. of mutation	Variants with MAF > 0.05	Gene region	Amino acid
Pre-VOC	1,175–2,275	1,100	1	C1191T	ORF1ab(nsp2)	P309L
	3,350–4,875	1,525	1	G4184A	ORF1ab(nsp3)	G1307S
	4,950–5,375	425	1	C5184T	ORF1ab(nsp3)	P1640L
	6,500–8,400	1900	2	C7124T, G8393A	ORF1ab(nsp3)	P2287S,A2710T
	8,650–8,885	235	–	–	ORF1ab(nsp4)	
	11,950–13,225	1,275	1	C12880T	ORF1ab(nsp7)	4205I
	13,400–13,650	250	–	–	ORF1ab(nsp12)	
	14,250–15,200	950	–	–	ORF1ab(nsp12)	
	16,350–16,550	200	1	C16466T	ORF1ab(nsp13)	P5401L
	16,850–19,550	2,700	2	C17410T	ORF1ab(nsp13)	R5716C
				A18163G	ORF1ab(nsp14)	I5967V
	20,575–21,475	900	–	–	ORF1ab-S	
Alpha	500–1950	1,450	1	C913T	ORF1ab(nsp2)	216S
	2,775–7,625	4,850	7	C3037T, A5041G, C5388A,T5952G, C5986T, T6954C, G7560A	ORF1ab(nsp3)	924F,1592Q,A1708D,L1896W,1907F,I2230T,R2432K
	8,075–10,775	2,700	3	G8131T, T9853C, C10450T	ORF1ab(nsp3-5)	K2622N,3196D,3395P
Beta	11,175–11,950	775	2	G11417T, G11540T	ORF1ab(nsp6)	V3718F,V3759F
	18,725–18,750	25	–	–	ORF1ab(nsp14)	
	21,525–22,275	750	1	C21714T	S	T51I
Delta	1,275–1,300	25	–	–	ORF1ab(nsp2)	
	7,050–7,250	200	1	C7124T	ORF1ab(nsp3)	P2287S
	9,200–9,275	75	–	–	ORF1ab(nsp4)	
	20,200–20,250	50	1	A20262G	ORF1ab(nsp15)	6,666 L
	20,400–20,475	75	–	–	ORF1ab(nsp15)	
	21,125–23,100	1975	7	C21618G, C21846T, G21987A, C22227T, T22917G, C22995A, G23012A	S	T19R,T95I,G142D,A222V,L452R,T478K,E484K
	25,675–26,575	900	1	C26054A	ORF3a	T221K
	26,800–28,700	1900	6	T27638C, C27739T, C27752T,C27874T, A28271T, A28461G	ORF7a,Intergenic &N	V82A,L116F,T120I,Intergenic,Intergenic,N:D63G
BA.1	6,025–6,325	300	–	–	ORF1ab(nsp3)	
	10,825–11,725	900	1	G11291A	ORF1ab(nsp5)	G3676S
	11,975–12,200	225	–	–	ORF1ab(nsp6)	
	18,425–18,475	50	–	–	ORF1ab(nsp14)	
	21,300–22,600	1,300	7	C21595T, C21762T, C21846T, G21987A, T22200G, G22578A, G22599A	S	11 V,A67V,T95I,G142D,V213G,G339D,R346K
	23,775–24,275	500	2	C23854A, C24130A	S	N764K,N856K
				C24130A	S	N856K
	25,600–25,625	25	–	–	ORF3a	
	26,500–26,600	100	2	A26530G	M	D3G
				C26577G	M	Q19E
	28,175–28,275	100	1	A28271T	Intergenic	
BA.2	8,600–8,700	100		–	ORF1ab(nsp4)	
Recombinant	500–2,275	1775	1	T670G	ORF1ab(nsp1)	S135R
	3,125–26,875	**23,750**	42	G4184A, G4184A, C4321T, T5386G, G8393A, C9344T, A9424G, C9534T, C9866T, G10447A, C10449A, G11291A, C12880T, C15714T, T16342C, C17410T, A18163G, C19955T, A20055G, C21618G, G21987A, T22200G, C22674T,T22679C C22686T, A22688G, G22775A, A22786C, G22813T, T22917G, G22992A, C22995A, A23013C, A23040G,A23055G A23063T, T23075C, C25000T, C25416T, C26060T, C26270T, C26577G, C26858T	ORF1ab(nsp3)-M	ORF1ab:G1307S, 1352A, 1707A, A2710T, L3027F, 3,050 V, T3090I, L3201F, 3394R, P3395H, G3676S, 4205I, 5,150 L S5360P, R5716C, I5967V, T6564I, 6597E, S:T19R, G142D, V213G, S371F, S373P, S375F, T376A, D405N, R408S, K417N, L452R, S477N, T478K, E484A, Q493R, Q498R, N501Y, H505Y, 1146D, ORF3a:8F, T223I, E:T9I, M:Q19E, 112F
	27,900–29,225	1,325	4	C28311T, G28881T, G28882A, G28883C	N	P13L, R203M, 203R, G204R

### Evaluation in the validation cohort highlights the diversity of SARS-CoV-2 transmission and evolution across different lineages

Accumulation of mutations within the viral genome that altered virulence, infectivity, and transmissibility was the primary driving force of viral evolution. Although mutations unique to different populations worldwide are reported, yet a heterogeneous distribution of co-existing mutations in distinct geographic regions within a particular population, such as India, is also reported. Comparative analysis of mutational trends of the SARS-CoV-2 genomes from geographic regions will help us in understanding differential selection pressure acting on the virus (55). Therefore, we carried out a comparative analysis of our

**Figure 7:**
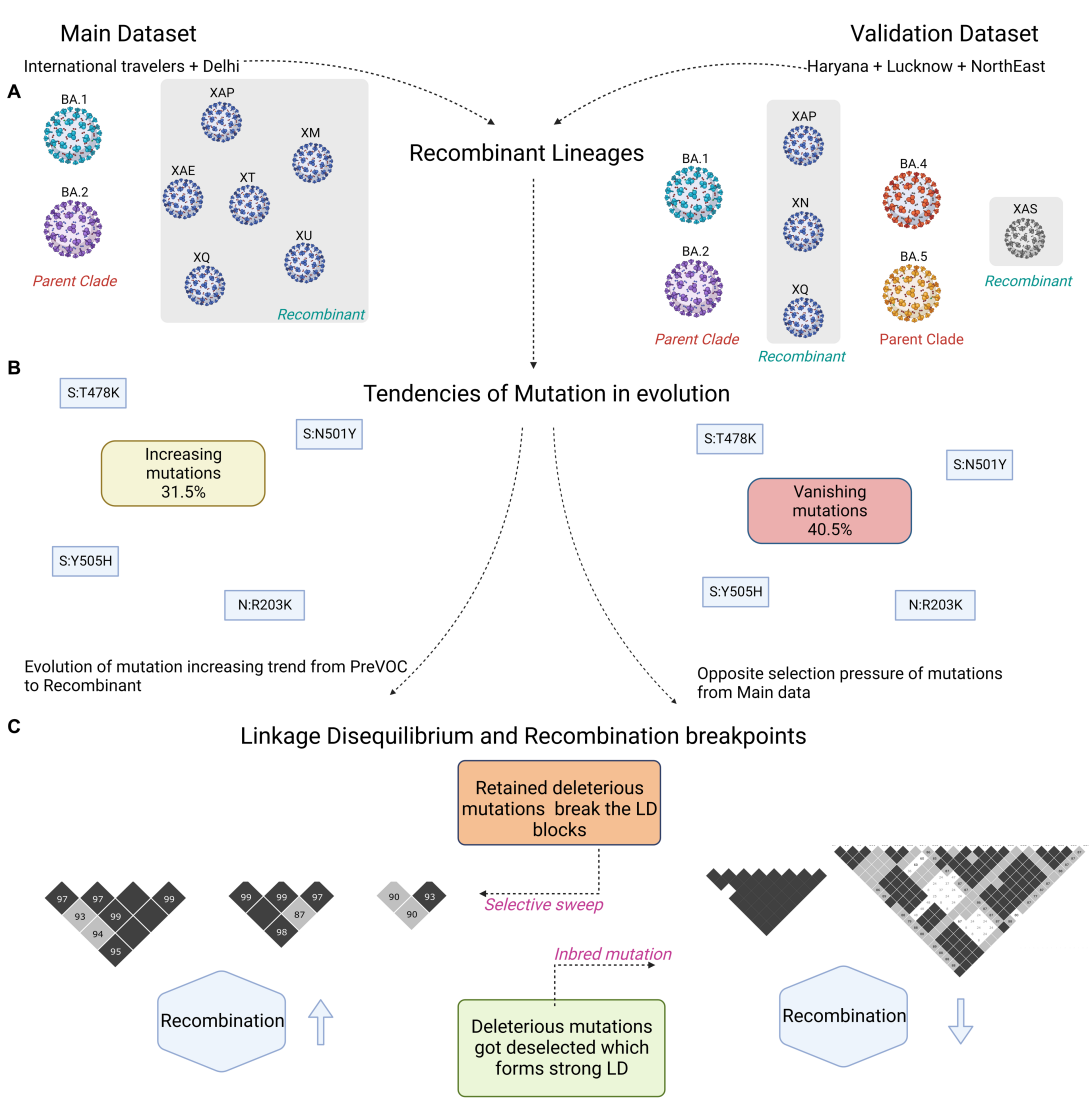
Summary figure capturing differences between the discovery and validation dataset. **(A)** Captures the Recombinant lineages and their parent clades. **(B)** Similar mutations harboring trends of increasing and vanishing in the two datasets. **(C)** Small vs. large LD block patterns.

“discovery” dataset with a “validation” dataset acquired through SARS-CoV-2 genome surveillance program in different states of India: MDU, Rohtak in Haryana [*n* = 808], CSIR-CDRI, Lucknow in Uttar Pradesh [*n* = 147] and CSIR-NEIST, Jorhat in Assam [*n* = 171] with the cutoff score of SARS-CoV-2 genome coverage of >50%. The sequences were analyzed for their lineage specificity: B.1 as Pre-VOC (*n* = 21), B.1.36 as Beta (*n* = 21), B.1.617.2 as Delta (*n* = 81), AY.* as Delta plus (*n* = 96), and Omicron grouped into four sub-lineages of BA.1 (*n* = 41), BA.2 (*n* = 617), BA.2.75 (*n* = 204), and BA.5 (*n* = 31). In this cohort, 13 samples were identified as Recombinants, 10 XN with one each of XAP, XAS, and XQ (Figure 7A; Supplementary Table S4). In the validation dataset, we identified a total of 1,663 mutations over the nine lineages, with an increase of mutational abundance from B.1.617.2 to BA.2.75, mainly from the ORF1ab region. BA.1 and BA.5 showed distorted patterns with higher abundance of mutations (Supplementary Figure S3A; Supplementary Table S5). Mutation trends of Delta and BA.2 carried a similar pattern, which was consistent with the discovery cohort findings. Analysis revealed higher presence of missense mutations followed by synonymous and deletion mutations (Supplementary Figure S3B). M gene exhibited a higher number of synonymous mutations, and ORF8 had the highest proportion of all mutation types (Supplementary Figure S3B), which aligned with the discovery dataset. Deletions were more prevalent in the 3’UTR compared to ORF7a of the discovery dataset. Furthermore, gene-wise mutational abundance showed a similar pattern; synonymous mutations were abundant in the ORF6 followed by ORF3a (Supplementary Figure S3C). For non-synonymous mutations, 5’UTR demonstrated more mutations in the Delta, whereas abundance in the Spike, E and ORF6 genes was observed for the Omicron lineages.

Interestingly, Recombinant had a low abundance of synonymous mutations, and in the non-synonymous, Spike, N and ORF6 demonstrated mutations with the trend followed in the Omicron lineages (Supplementary Figure S3C). Delving deeper into the 73 Recombinant mutations, 19 originated from Delta, three each from Pre-VOC and Beta, four from BA.2, five from BA.2.75, and the rest 39 mutations were from the BA.1 (Supplementary Figure S3D). Subsequently, the comparison of mutational dynamics across discovery and validation datasets strikingly demonstrated a distinct mutational percentage distribution among the five mutational trends (*spontaneously increasing*, *spontaneously decreasing*, *spontaneously vanishing*, *dynamically increasing*, and *dynamically decreasing*), except in the *dynamically vanishing* group (Figure 7B; Supplementary Figure S4). *Spontaneously increasing* mutations (31.5%) which showed the highest presence in discovery were replaced by the *spontaneously vanishing* mutations (40%) in the validation dataset (Supplementary Figure S4). However, most of the individual mutations were found overlapping between the discovery and validation datasets across the different mutational trends. Except, *dynamically decreasing* mutations of validation were identified as overlapping with the *spontaneously/dynamically increasing* in the discovery set, reflecting a completely opposite selection pressure on the mutations over the course of time in the distinct human host populations. Moreover, contrary to our discovery dataset, LD analysis in the validation cohort demonstrated an increase in the number and size of the LD blocks while evolving toward the Omicron (Figure 7C; Supplementary Figure S5). Contrary to the observation that the LD blocks dissipate down the lineages in the discovery data, the validation cohort LD analysis demonstrated an increase in the LD blocks after Beta till BA.2. Unlike the discovery, Pre-VOC in the validation cohort had a completely distinct pattern of haplotype blocks which did not get retained in the lineages after that (Supplementary Figure S5A).

The validation cohort portrayed a predominant presence of large LD blocks in the ORF1ab gene regions in the Pre-VOC and Beta; however, Delta onwards, blocks were reduced in size and were confined to the 5′ region of ORF1ab (~9-11 kb) (Supplementary Table S6). Interestingly, we observed a similar trend in the discovery cohort as well, where the LD blocks were seen from the Pre-VOC to Delta, completely vanished in BA.1 and BA.2, but arose in the Recombinant, confined to the 5′ region of ORF1ab. This highlighted the importance of ORF1ab in the SARS-CoV-2 recombination, particularly the 3’region of the gene. Additionally, strong LD blocks in the spike region were found in all the lineages except BA.1 and Recombinant in the validation cohort, dissimilar from the discovery data. It is important to note that LD blocks were significant and spanned comparatively larger gene regions in the BA.2 of the validation cohort, whereas it was completely contradictory in our discovery data with a small number and size of LD blocks in the BA.2. Furthermore, the N gene LD blocks with the mutations—N: R203M/203R/G204R were seen to be inherited till the Recombinants in discovery as well as the validation cohort, which showed that a higher degree of conservation was maintained in the SARS-CoV-2 N gene. Intriguingly, the validation cohort displayed a higher presence and retention of 3′ region LD blocks of SARS-CoV-2 genes, including ORF3a, M and ORF7a till BA.1, which was completely absent in the discovery cohort. Overall, the LD blocks increased in size and number in the validation cohort compared to the discovery, except in the 3′ region of the ORF1ab gene. The complete LD decay signal was very random from Pre-VOC to Recombinant (Supplementary Figure S6); however, regression coefficient showed a positive correlation for Pre-VOC similar to the discovery data, while a higher negative correlation in Beta (−0.0000197) followed by Recombinant (−0.0000125) (Supplementary Table S7). This was validated in the breakpoint data wherein the longest breakpoint was observed in the Pre-VOC followed by the Beta and Recombinant (Supplementary Table S7). Overall, the decay signal of BA.1 and BA.2 is in alignment with the discovery data with conservation of the 3’UTR region in BA.2.

## Discussion

SARS-CoV-2, similar to all RNA viruses, undergoes mutation and recombination as part of its evolutionary process. Recurrent mutations and recombination are crucial forces that let the virus adapt/evolve within its host microenvironment. Similarly, diverse recombination frequencies were seen in other distinct families of viruses. For instance, HIV is noted for the most frequent recombination events (56) followed by the positive-single strand RNA virus families such as coronaviridae, bromoviridae, and potyviridae (57, 58). Moreover, negative-single strand RNA viruses such as Influenza A are reported to have less frequent recombination events attributing to their segmented genomes (59, 60). It is also important to note that the time of infection, either persistent or acute, contributes to the recombination frequency as a host can be co-infected with different strains of the same virus during the course of time. In case of SARS-CoV-2, the initial emergence of Recombinants such as XA (inter-VOCs) and XE (hybrid of BA.1 and BA.2), WHO reported XBB as variant of interest (VOI) in January 2023, and recently, EG.5 with Initial Risk Evaluation in August 2023. Recombination events reported in the SARS-CoV-2 are both intervariant and within Omicron sub-lineages (61). Although the recombination lineages obtained in our data arose from BA.1 and BA.2, the mutational dynamics of Recombinants were not the same as their parent clades. This could possibly be resultant of the general mechanism of RNA viruses, whereby they undergo selective pressure for elimination of detrimental mutations from parental clades and keeping only those that are advantageous (62) for their sustainability within the host. Additionally, an increase and decrease in the frequency of mutations could also occur due to chance events (63), which has been reported wherein limited number of viruses established a new population during transmission as might have happened during recombination. The dilution of the mutations in Recombinants can plausibly explain their inability to propagate or transmit and infect at faster rates as observed for the previous lineages of Delta and Omicron. Among the retained mutations, ten mutations that originated from the Delta were responsible for enhanced fusogenicity, infectivity, and were majorly involved in increasing pathogenicity (64, 65), yet their presence with differential frequencies in the Recombinants might have ameliorated their desired effects. The mutations such as S (G142D) and S (T95I) played significant driver role in the evolutionary process and affected the viral load during Delta propagation (66, 67).

Delving deeper into the adaptive and deleterious mutations during SARS-CoV-2 evolution, represented by the six categories of mutations as in Figure 3A, we found that *spontaneously increasing* mutations were most prominent with a higher percentage (31.5%) whereas *dynamically increasing* displayed low presence (3.4%). Notably, these progressively increasing mutations were long accumulating mutations since majority of them were present in low frequency in Pre-VOC and substantially resurfaced in the Omicron and Recombinants. Mutations, such as ORF1ab: T3255I, are reported to be responsible for symptomatic disease outcome and changed interactome toward avoidance of antiviral response, improving viral spread and infectivity as seen in the Omicron (68), while ORF3a: T223I, ORF1a: P3395H and ORF1ab: I5967V were retained till BA.2 and BA.3, but the functional significance of such mutations are still unknown (69, 70).

The *dynamically increasing* mutations (Figure 3H) although low in percentage, yet, important as these mutations have undergone selection and de-selection by the virus across the lineages at different time points and tend to increase during evolution. These diverse trends of mutations played a major role in the observed LD forming haplo-blocks, where we found longer blocks in the Pre-VOC and blocks that tended to shrink in the Omicron lineages. Supposedly, sudden bursts of mutations might have decreased the LD strength and created regions of weak LD which would have been beneficial for giving rise to Recombinants as observed due to the likelihood of recombination in the Omicron sub-lineages. Recombination rates can evolve especially when there is weak negative epistasis among favorable alleles. Weak negative epistasis implied that the fitness of a genotype with several advantageous alleles was slightly less than would be expected from the product of the fitness of each allele measured separately (71). Thus, selection generates negative LD between the favorable alleles. During recombination, this disequilibrium is broken and hence increased variance in the positive selection. Resultantly, recombination processes disrupt LD and manage to alter the variants-associated sites (72). Moreover, decay of the LD was complimented by the haplo-blocks in the lineages, wherein LD decay was observed from BA.1 lineage with expectation of recombination events. Evidence suggests that recombination breakpoints were more prominent in the Spike gene and three times higher in the 3′ end of the Spike (73, 74). Similarly, we observed breakpoints in the 3’end of the Spike only in the BA.1, spanning between the genomic positions of 23,775–24,275, which contained S: N764K and S: N856K mutations. Interestingly, these mutations were not in strong LD with other upstream and downstream mutations forming haploblocks. Although the frequency of recombination breakpoints toward 5′ end of the Spike region was reportedly less (74, 75), we found overlapping breakpoints in the Spike’s 5′ region in Delta (genome position 21,225–23,100), BA.1 (genome position 21,300–22,600), and Recombinant (genome position 3,125–26,875), which might be associated with common mutation S:G142D in all the three lineages, wherein it is responsible for immune evasion properties.^1^ Mutation, S:T95I common in the Delta and BA.1, S: V213G, S: L452R, S: T478K common in the Delta and Recombinant, were known for its immune evasion, fusogenicity, increased viral replication, and binding affinity for Spike-ACE2 receptor (21, 43, 76). The identification of recombination breakpoints spanning various lineages highlighted the intricate interplay of recombination dynamics among the different viral lineages, providing valuable insights into the mechanisms driving viral evolution.

Finally, toward functional relevance interpretation of the findings from the discovery data, it was compared with an in-house independent validation dataset. The comparison of these two datasets not only enhances the methodological rigor but also emphasizes the relevance of our findings within the context of continued SARS-CoV-2 genomic Surveillance in the Indian population. Additionally to highlight the uniqueness of the study, we have detected the recombination through the lens of mutations of SARS-CoV-2 whole genome data, which paves the way for potential advancements in pathogen genomics in identifying recombination events from genome data. It is essential to note that the timeline of samples for discovery and validation might have played a key role in forming strong haplo-blocks and recombination breakpoints, especially for the Omicron variants, where strong and lengthy haplo-blocks for validation data was observed which was in variance with the discovery data. The timeline of collection of BA.1 samples for the discovery cohort ranged from December 2021 to January 2022, whereas for the validation cohort, it was January 2022. Similarly, BA.2 timeline extended from January to June 2022 for the discovery and January to August 2022 for the validation dataset (Supplementary Tables S1, S5). Furthermore, the relevance of finding the emergence of Recombinant variants subsequent to the Omicron lineages as revealed through LD, LD decay, and recombination breakpoints provides a valuable insight into the evolutionary dynamics, driven by mutations or recombination events. This contributed directly comprehending the origin and evolution of novel SARS-CoV-2 variants, shedding light on the epidemiological aspects and infectivity. Additionally, the geographic diversity of the validation data [Rohtak (Haryana), Lucknow (Uttar Pradesh) and Jorhat (Assam)] strengthened the LD blocks and reduced the presence of recombination breakpoints, which was also demonstrated by another study where different Association of Southeast Asian Nations (ASEAN) populations displayed little to no recombination (72), confirming different selection pressure for survival and evolution in Omicron lineages. These findings have the potential to play a crucial role in understanding the virus’s spread, with implications for epidemic or pandemic scenarios. Further studies are needed to evaluate the linkage disequilibrium and the impact on fitness factors for Omicron lineages.

## Conclusion

This study highlights mutational diversity and abundance evaluation across seven broadly categorized lineages of SARS-CoV-2 since the Pre-VOC times, identifying the trends of mutations harbored by the Recombinants and their functional relevance. Mutational trend analysis through different lineages revealed that *spontaneously increasing* mutations retained in Recombinants were majorly associated with decreased SARS-CoV-2 infectivity, replication, and disease severity. The *dynamically vanishing* and *decreasing* mutations were from the Beta and Delta (reportedly elevated SARS-CoV-2 infection), which were eliminated by the virus during evolution into Omicron and Recombinants. Small LD blocks in BA.1 and BA.2 might have led to generation of multiple lineages within Omicron as well as emergence of the Recombinants. LD decay patterns were observed from BA.1 and were corroborated with breakpoint analysis. These findings provided insights into the mechanisms of evolution of SARS-CoV-2 through recombination across several lineages of different time points.

## Data availability statement

The datasets presented in this study can be found in GISAID repository. The GISAID accession numbers are provided in the Supplementary material.

## Ethics statement

The studies involving humans were approved by the Institutional Ethics Committee of CSIR-Institute of Genomics and Integrative Biology, under the approval number CSIR-IGIB/IHEC/2020-21/01. The studies were conducted in accordance with the local legislation and institutional requirements. Written informed consent for participation in this study was provided by the participants’ legal guardians/next of kin.

## Author contributions

VR: Formal analysis, Investigation, Methodology, Visualization, Writing – original draft. US: Conceptualization, Data curation, Visualization, Writing – original draft, Writing – review & editing. MK: Formal analysis, Methodology, Visualization, Writing – original draft. AS: Writing – review & editing. PM: Methodology, Writing – review & editing. RS: Resources, Writing – review & editing. PB: Resources, Writing – review & editing. NC: Resources, Writing – review & editing. RP: Conceptualization, Funding acquisition, Investigation, Project administration, Supervision, Visualization, Writing – review & editing.

## Footnotes

^1^ https://doi.org/10.1101/2021.09.12.21263475/medrxiv
