# Supplementary material for: Unraveling the genetic evolution of SARS-CoV-2 Recombinants using mutational dynamics across the different lineages
Source: Front Med (Lausanne). 2024 Jan 15;10:1294699. doi: 10.3389/fmed.2023.1294699 (PMC10823376; doi:10.3389/fmed.2023.1294699)
Supplement: Supplementary file 8 [file Data_Sheet_1.docx]

***Supplementary Material***

**Unraveling the genetic evolution of SARS-CoV-2 Recombinants using mutational dynamics across the different lineages**

Varsha Ravi^1,$^, Uzma Shamim^1,$,*^, Md Abuzar Khan^1^, Aparna Swaminathan^1^, Pallavi Mishra^1^, Rajender Singh^2^, Pankaj Bharali^3^, Nar Singh Chauhan^4^, Rajesh Pandey^1,5,*^

^1^Division of Immunology and Infectious Disease Biology, INtegrative GENomics of HOst-PathogEn (INGEN-HOPE) laboratory, CSIR-Institute of Genomics and Integrative Biology (CSIR-IGIB), Mall Road, Delhi-110007, India.

^2^CSIR-Central Drug Research Institute, (CSIR-CDRI), Lucknow, India.

^3^CSIR-North East Institute of Science and Technology (CSIR-NEIST), Pulibor, Jorhat, Assam-785006, India.

^4^Department of Biochemistry, Maharshi Dayanand University, Rohtak, India.

^5^Academy of Scientific and Innovative Research (AcSIR), Ghaziabad-201002, India.

^$^Equal contribution

^*^Co-corresponding Authors

**Contact Details**

Rajesh Pandey, PhD

Principal Scientist,

INtegrative GENomics of HOst-PathogEn (INGEN-HOPE) laboratory,

CSIR-Institute of Genomics and Integrative Biology (CSIR-IGIB),

North Campus, Near Jubilee Hall, Mall Road, Delhi-110007, India.

Email: rajeshp@igib.in; rajesh.p@igib.res.in; Tel.: 011-27002200 (Ext. 254)


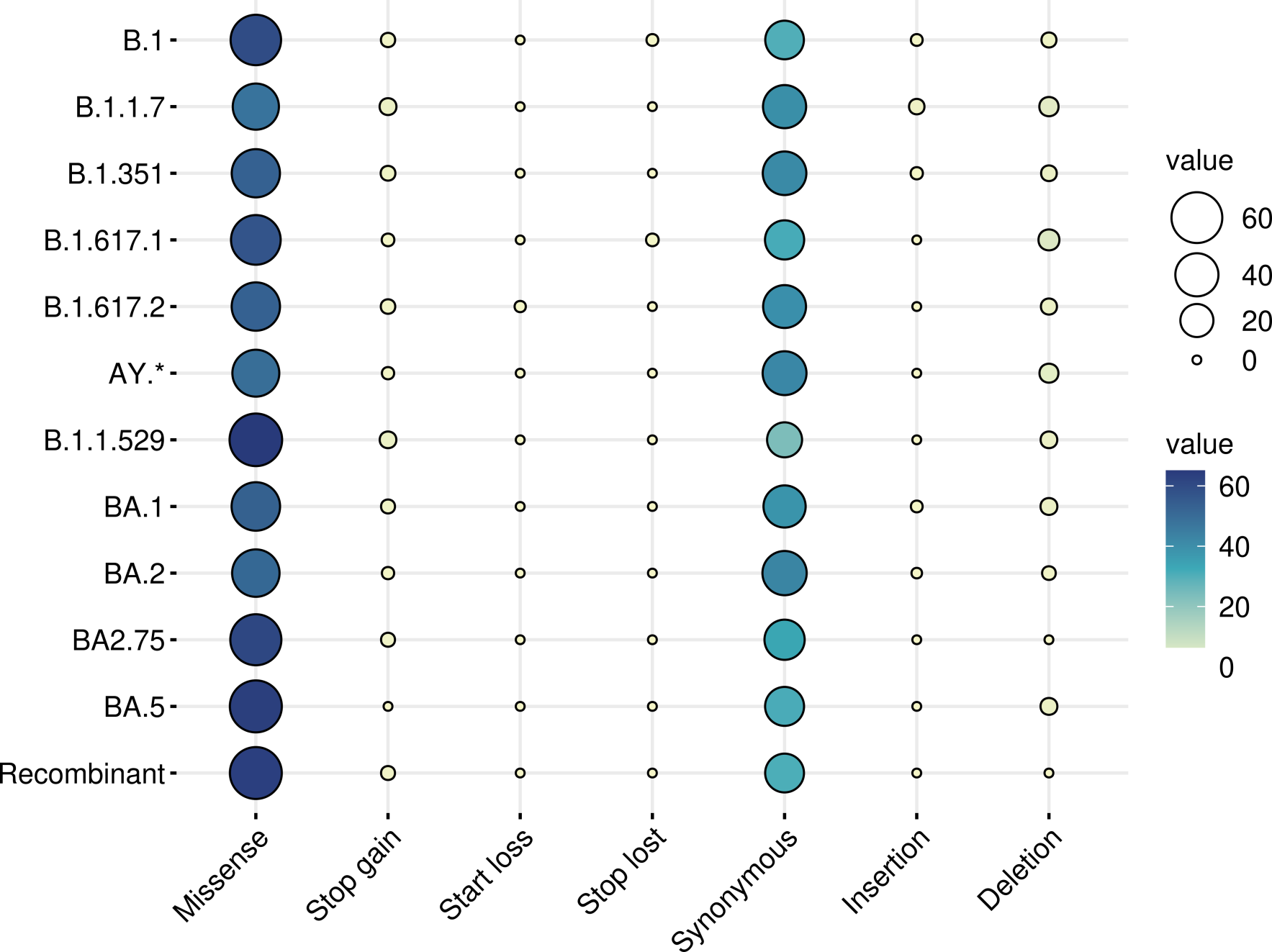


**Supplementary Figure S1**: Types of mutations and their correlation with respect to lineages from the Pre-VOC (B.1) to the Recombinant in the discovery data.


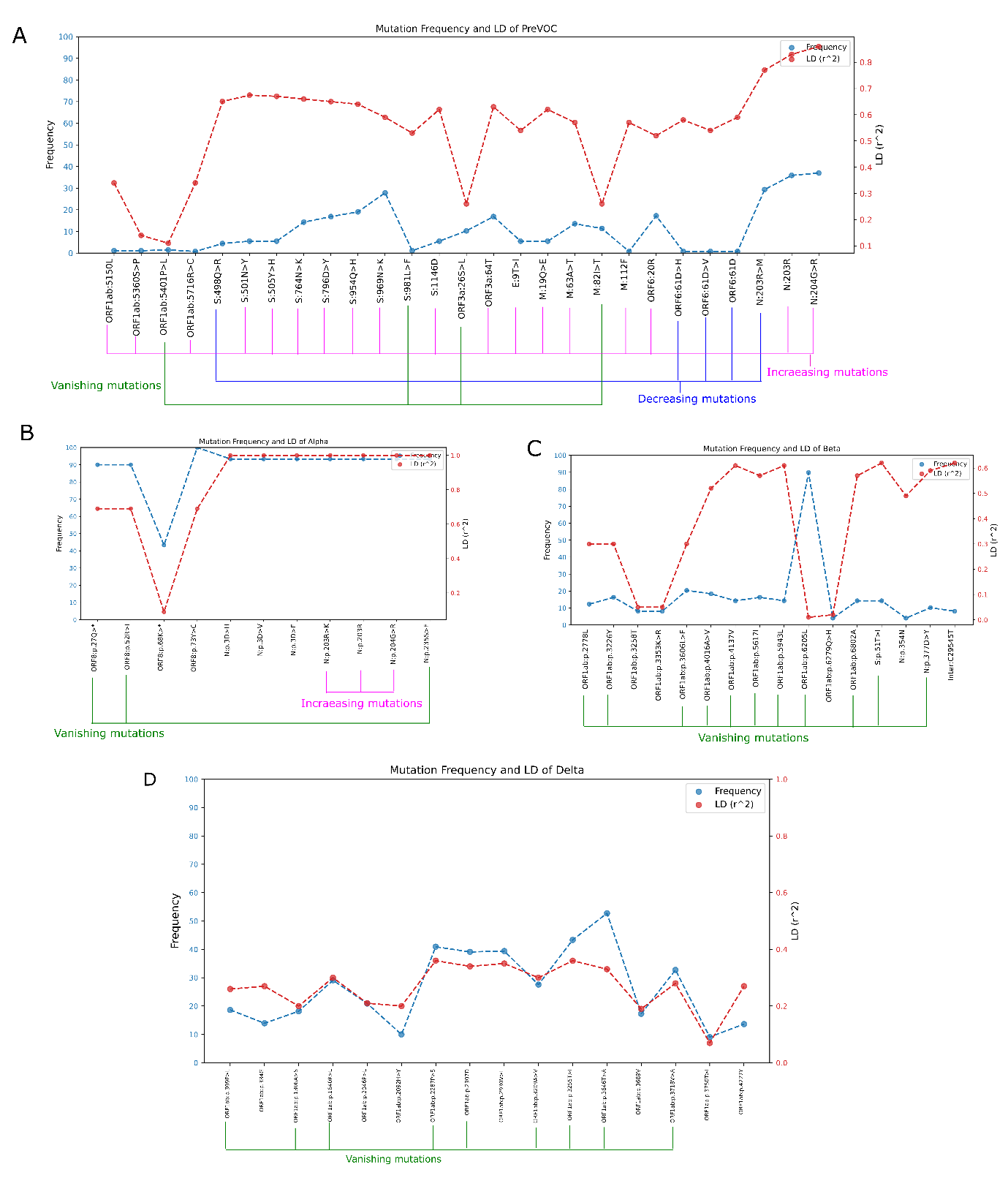


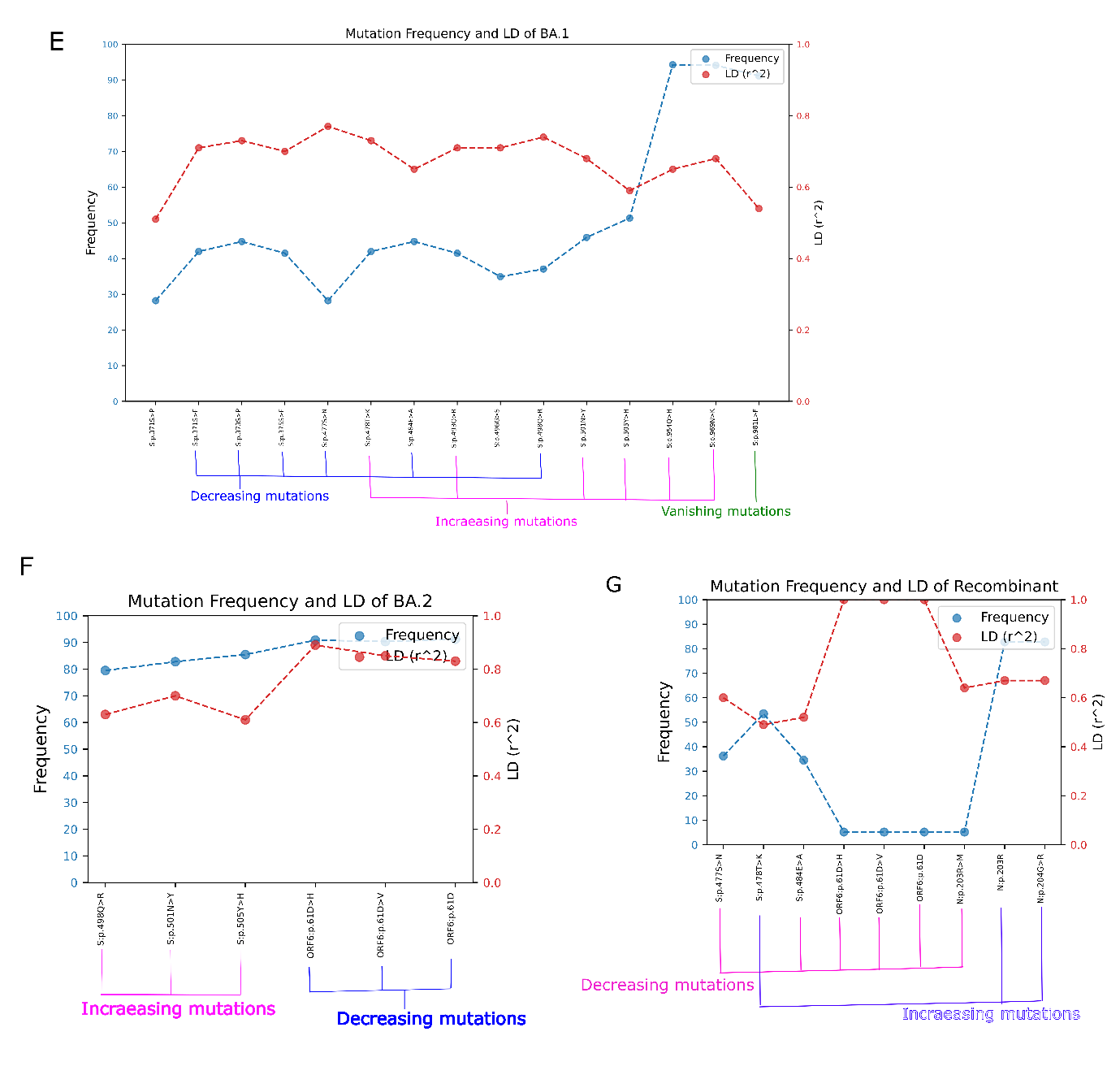


**Supplementary Figure S2**: Mutation Frequency and LD (r2) trend plots depicting increasing, decreasing and vanishing mutations.


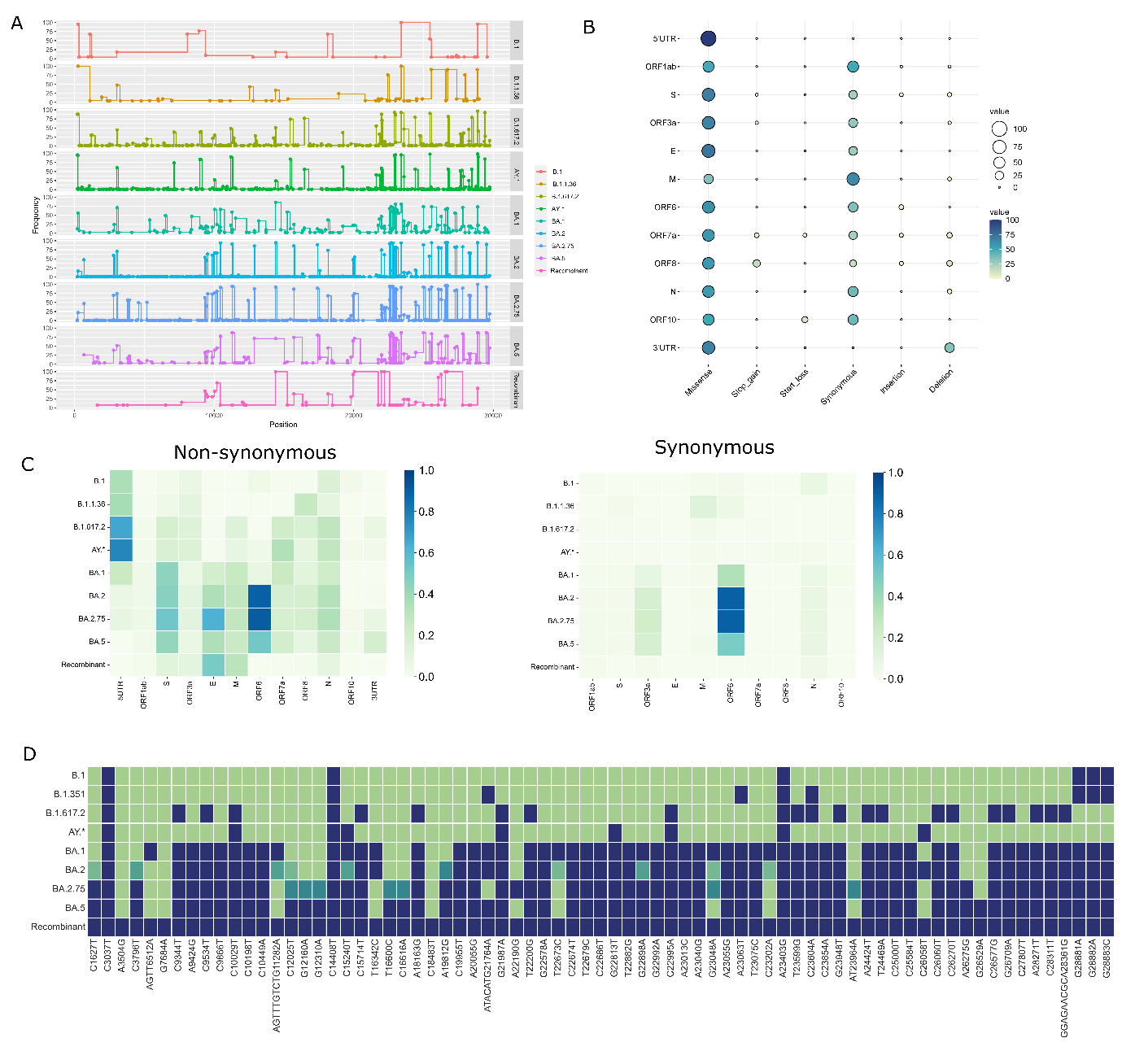


**Supplementary Figure S3**: Mutational dynamics of validation data. **(A)** Mutational trends of the Pre-VOC to Recombinant. **(B)** Types of mutations and their affluence in Gene-region. **(C)** Mutational abundance in Gene-regions with respect to lineages. **(D)** Heat map represents Recombinant mutations and respective mutation frequency in other lineages.


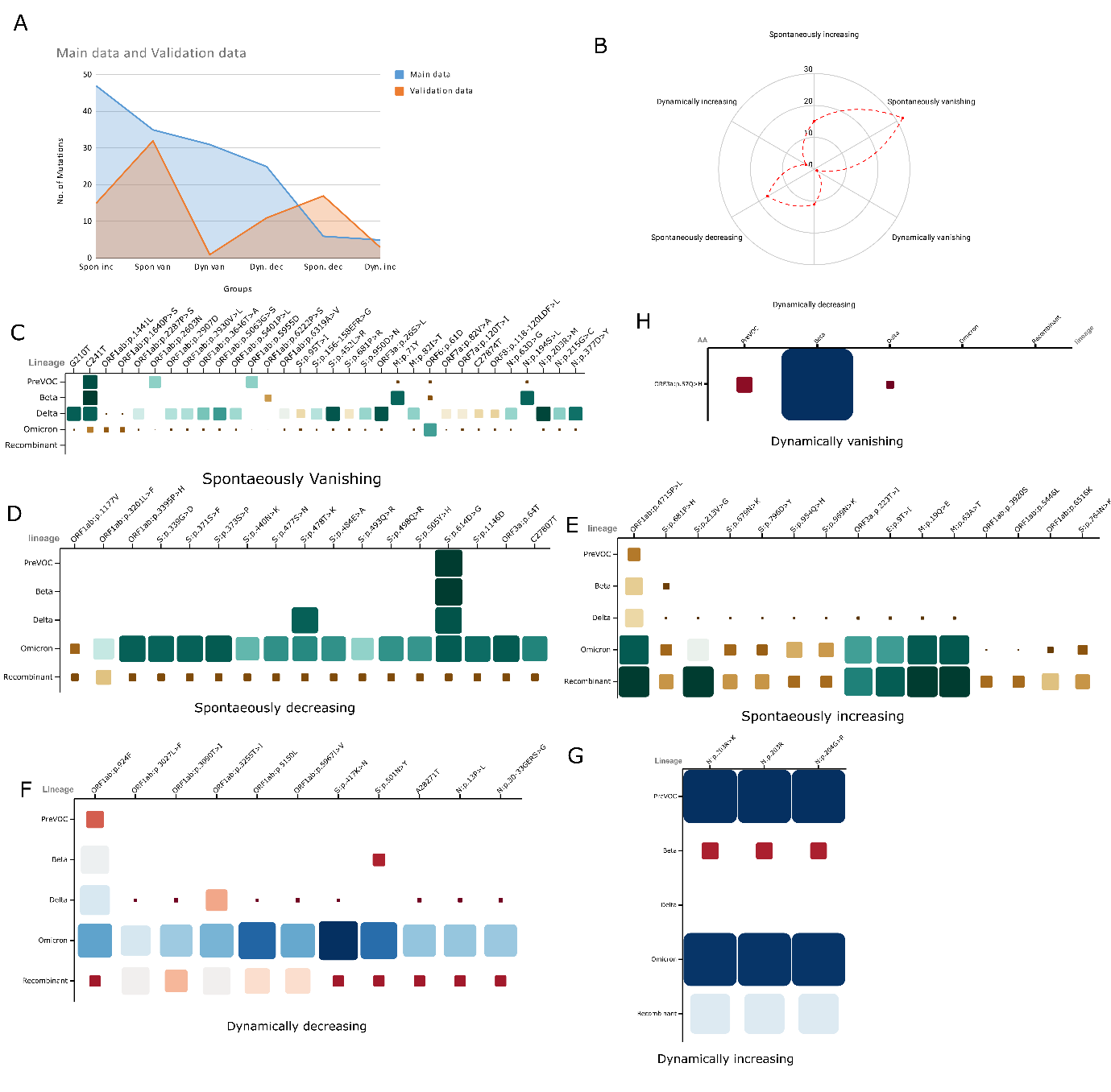


**Supplementary Figure S4**: Trends of mutations across lineages in validation cohort. **(A)** Comparison of mutation groups between discovery and validation data. **(B)** Spider plot depicts the proportion of mutations in the groups for validation data. **(C-E)** Matrix plot for *Spontaneously vanishing, decreasing* and *increasing* groups. **(F-H)** Matrix plot for *Dynamically decreasing, increasing* and *vanishing* groups.


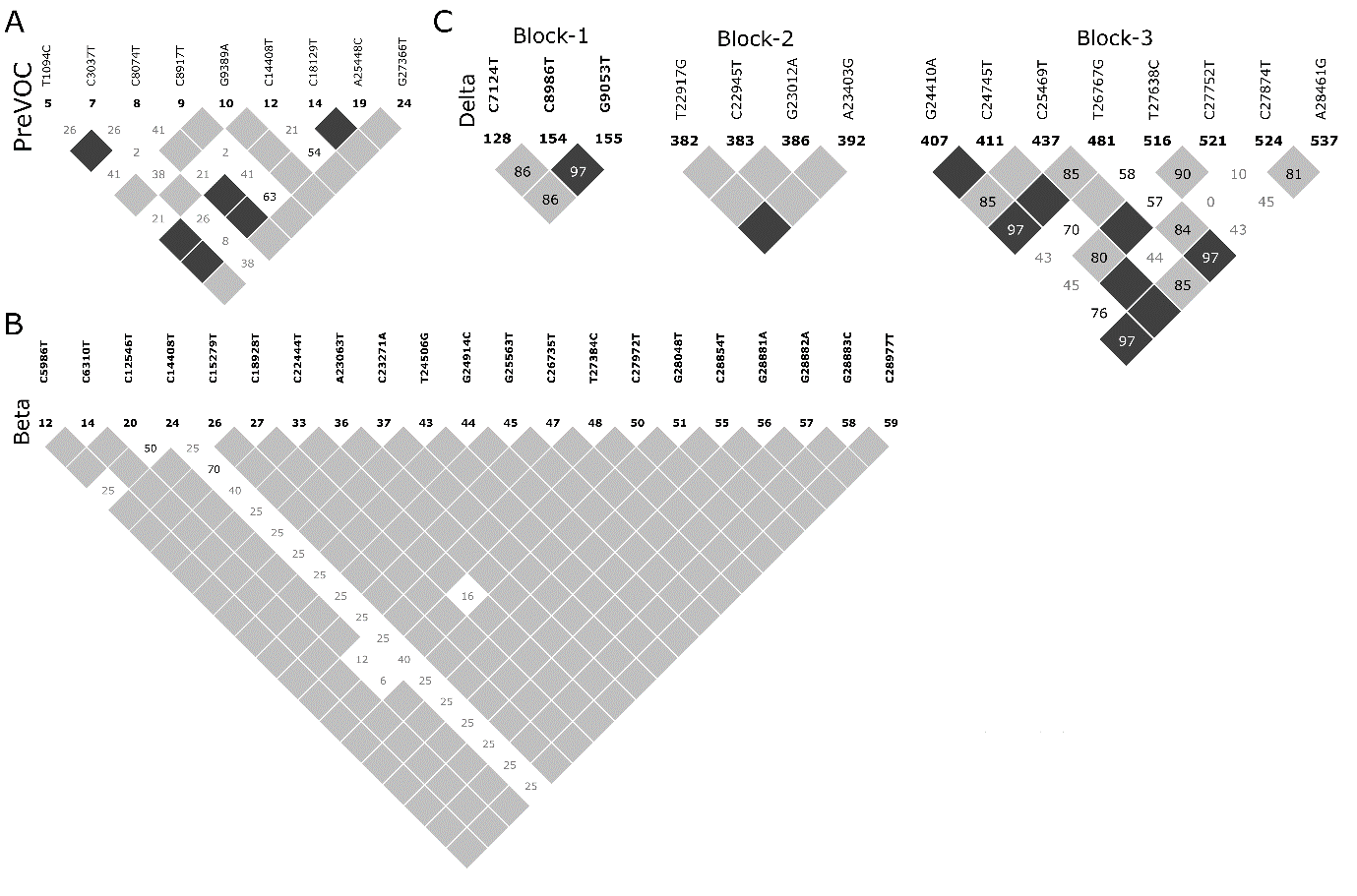


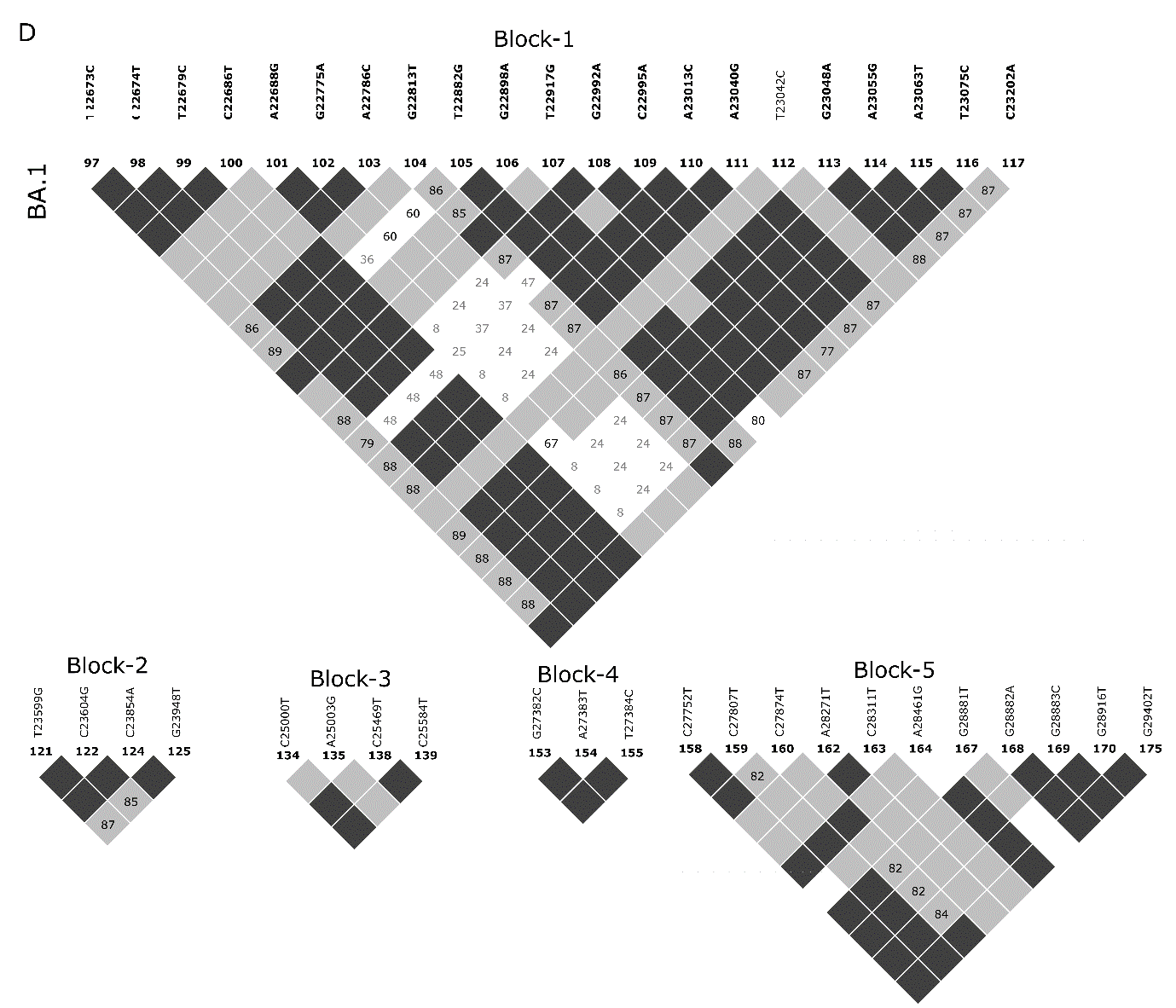


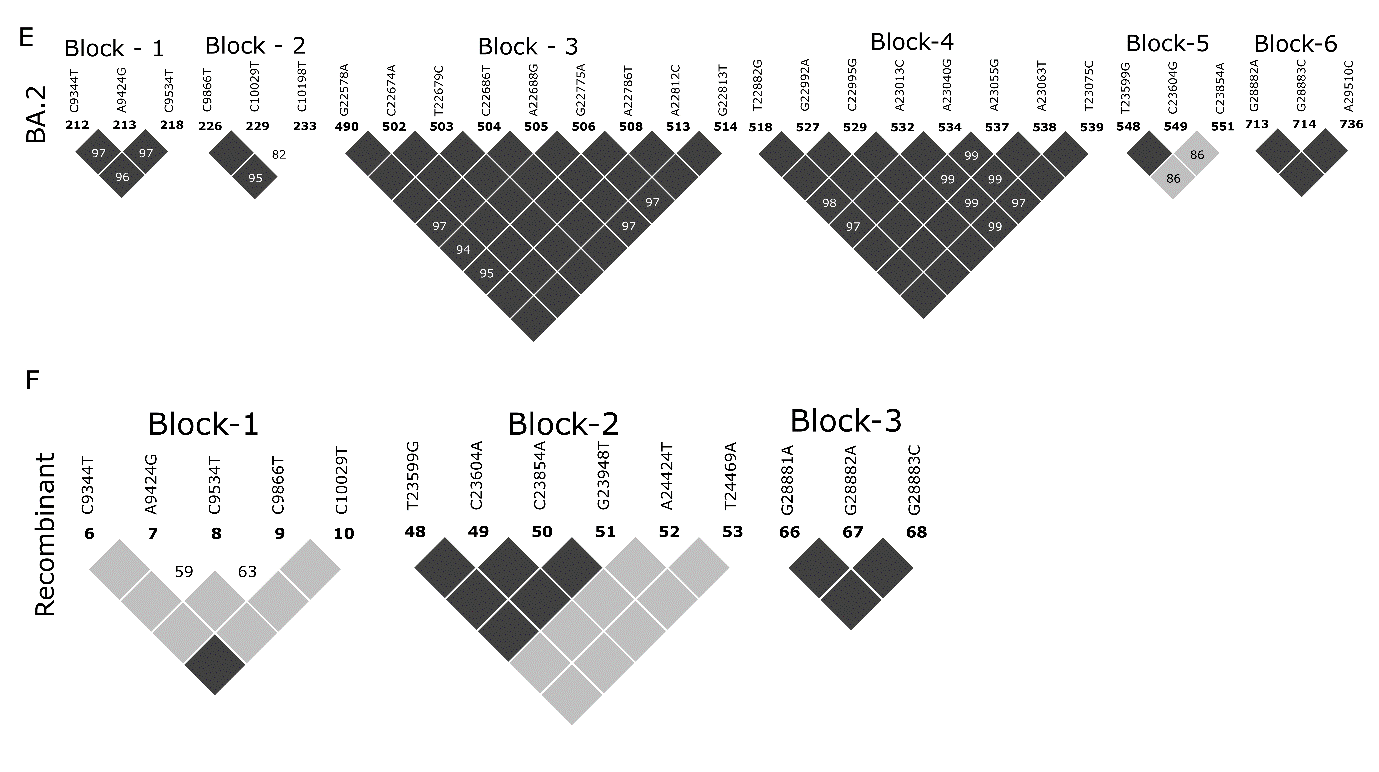


**Supplementary Figure S5**: Linkage Disequilibrium and Haplotype blocks of Validation data. (A-F) Pre-VOC to Recombinant.


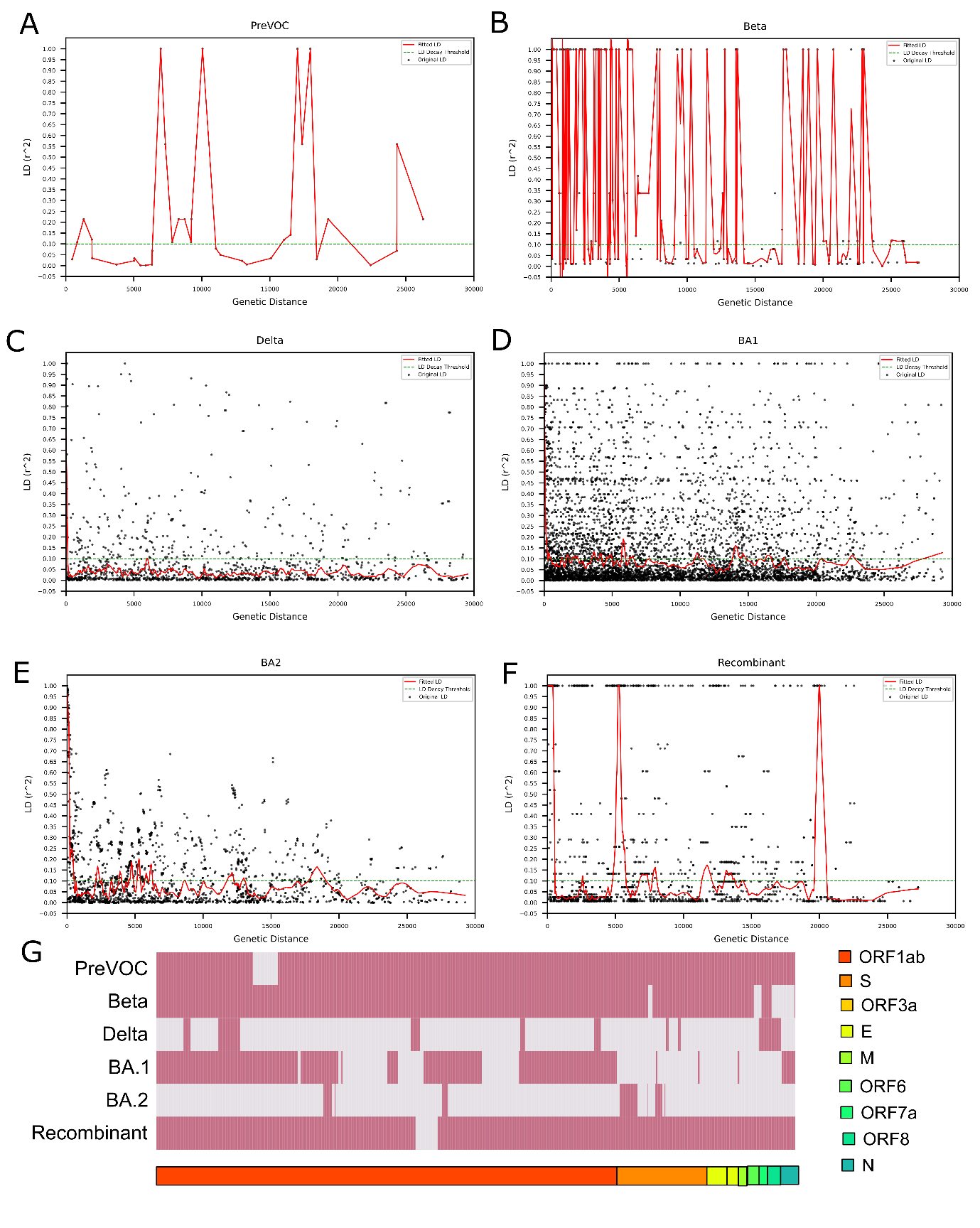


**Supplementary Figure 6**: LD decay for the Pre-VOC to Recombinant and breakpoint. (A-F) Pre-VOC, Beta, Delta, BA.1, BA.2, and Recombinant. (G) Heat map demonstrated the breakpoint from the Pre-VOC to the Recombinant. **Pink colour signifies breakpoint in that position.
